# Adaptation and validation of an adult patient classification instrument with emphasis on the family dimension

**DOI:** 10.1590/0034-7167-2022-0530

**Published:** 2023-03-27

**Authors:** Ariane Polidoro Dini, Jamilly dos Santos Okabe, Stephanie Ziare Kalvan, Carla Simplicio, Renata Cristina Gasparino

**Affiliations:** I Universidade Estadual de Campinas. Campinas, São Paulo, Brazil

**Keywords:** Validation Studies, Factor Analysis, Hospital Organization and Administration, Patient-Centered Care, Family., Estudios de Validación, Análisis Factorial, Organización y Administración, Atención Dirigida al Paciente, Red Familiar., Estudos de Validação, Análise Fatorial, Organização e Administração, Assistência Centrada no Paciente, Rede Familiar.

## Abstract

**Objectives:**

to adapt and validate an instrument for classifying adult patients that emphasizes the family support network in the demand for nursing care.

**Methods:**

methodological study, carried out in three phases: adaptation of an instrument considering the reality of adult patients; content validation with seven experts and assessment of measurement properties (construct validity and internal consistency) with 781 hospitalized patients.

**Results:**

in content validation, the indicators reached the values established for the Content Validity Index (0.85-1.00). In the confirmatory factor analysis, the 11 indicators were distributed in three domains and presented average variance extracted and factor loading greater than 0.5. Composite reliability was greater than 0.7.

**Conclusions:**

the present study adapted and made available, with evidence of validity and reliability, an instrument for classifying adult patients that considers the family support network in the demand for nursing care.

## RESUMO

**Objetivos:**

adaptar e validar um instrumento para classificação de pacientes adultos que enfatiza a rede de suporte familiar na demanda de cuidados de enfermagem.

**Métodos:**

estudo metodológico, realizado em três fases: adaptação de um instrumento considerando a realidade de pacientes adultos; validação de conteúdo com sete especialistas e avaliação das propriedades de medida (validade de construto e consistência interna) com 781 pacientes internados.

**Resultados:**

na validação de conteúdo, os indicadores alcançaram os valores estabelecidos para o Índice de Validade de Conteúdo (0,85-1,00). Na análise fatorial confirmatória, os 11 indicadores foram distribuídos em três domínios e apresentaram variância média extraída e carga fatorial superiores a 0,5. A confiabilidade composta foi superior a 0,7.

**Conclusões:**

o presente estudo adaptou e disponibilizou, com evidências de validade e confiabilidade, um instrumento para classificação de pacientes adultos que considera a rede de suporte familiar na demanda de cuidados de enfermagem.

## RESUMEN

**Objetivos:**

adaptar y validar un instrumento para clasificación de pacientes adultos que enfatiza la red de apoyo familiar en la demanda de cuidados de enfermería.

**Métodos:**

estudio metodológico, realizado en tres fases: adaptación de un instrumento considerando la realidad de pacientes adultos; validez de contenido con siete especialistas y evaluación de las propiedades de medida (validez de constructo y consistencia interna) con 781 pacientes internados.

**Resultados:**

en la validez de contenido, los indicadores alcanzaron los valores establecidos para el Índice de Validez de Contenido (0,85-1,00). En el análisis factorial confirmatorio, los 11 indicadores fueron distribuidos en tres dominios y presentaron desviación mediana extraída y carga factorial superiores a 0,5. La confiabilidad compuesta fue superior a 0,7.

**Conclusiones:**

el presente estudio adaptó y proveyó, con evidencias de validez y confiabilidad, un instrumento para clasificación de pacientes adultos que considera la red de apoyo familiar en la demanda de cuidados de enfermería.

## INTRODUCTION

Nursing management is challenged daily by demands that, for sustainability and safe care, require a great deal of involvement from nurses in administrative, financial and economic issues, in addition to mastering modern tools for the development of managerial competences, such as leadership, flexibility, proactivity, among others^(1-3)^.

Physical, material, technological and financial resources, although essential for the achievement of quality care, are subordinated to the real needs of users, as well as the knowledge of professionals about the quantity and optimization of the use of these resources^(2-3)^.

In surveying the real needs of patients, the search for instruments that measure objectively and practically with a focus on nursing work and the implementation of efficient information systems have undeniable applications in promoting strategies that enable the balance between demand and supply of a safe care^(4-12)^. In this direction, the Patient Classification System (PCS) is a tool that stands out for helping management in the dimensioning of the nursing staff^(2,4-7)^.

The PCS provides information and statistical data that help decision making and problem solving in the management of financial, human and material resources^(1-12)^. Thus, the evaluation of the measurement properties of instruments for this purpose is of paramount importance so that the results can safely support nursing management^(5)^.

In view of the particularities of the pediatric clientele and the constant presence of a mother or accompanying family member during hospitalizations, the Pediatric Patient Classification Instrument (PPCI) was built and validated, which included care indicators related to the patient, his family and some hospital routine procedures^(13)^.

Today, the presence of family members and companions is common for adult patients, many of whom were previously healthy and underwent surgical treatment with early discharge^(14)^; in this sense, the patient classification instruments must progress with the care scenario to safely and reliably support the nursing management practice^(4-5,7,12)^. Considering this context, the question that guided the present study was: Does the adaptation of the Pediatric Patient Classification Instrument for the classification of adult patients have evidence of validity and reliability to be used in managerial nursing practice?

## OBJECTIVES

To adapt and validate an instrument for classifying adult patients that emphasizes the family support network in the demand for nursing care.

## METHODS

### Ethical aspects

The study was approved by the Research Ethics Committee of the University where it took place.

### Design, study location and period

Methodological study, carried out in a general, public, and teaching hospital, a reference for the care of patients in the interior of the state of São Paulo and coming from the Unified Health System (SUS).

The research was developed in three phases, which took place between November 2016 and November 2018: 1) Adaptation of the instrument^(13)^; 2) Content validation^(15)^; and 3) Evaluation of measurement properties^(16-18)^.

### Study protocol

In phase 1, a bibliographic survey was carried out, based on the current legislation for the dimensioning of nursing staff^(12)^, references for the validation of measurement instruments^(14-18)^, family-centered care and adult and elderly patients^(19-24)^. In the analysis of the literature, the Pediatric Patient Classification Instrument (PPCI) was found, which includes the family support network in the demand for nursing care, through 11 indicators distributed in three evaluation domains: “Family”, “Patient” and “Therapeutic procedures”^(13)^. Thus, with the author’s authorization, the content of the PPCI^(13)^ was adapted to include adult and elderly patients.

Still at this stage, the authors also adapted the response scale to the routine care of adult and elderly patients. The classification of patients in the category of care was determined according to the current legislation for the dimensioning of nursing staff, that is, minimal, intermediate, high dependency, semi-intensive and intensive care^(12)^.

In phase 2, the adapted instrument called the Adult Patient Classification Instrument (APCI) had its content evaluated by a group of specialists^(14-15)^. Participants analyzed the relevance and clarity of each of the indicators, using a four-point Likert scale: (1) Not relevant or not clear for assessing the demand for nursing care; (2) Needs major revision to be relevant or provide clarity in assessing nursing care demand; (3) Needs minor revision to be relevant or provide clarity in assessing nursing care demand; or (4) Relevant and representative in the assessment of nursing care demand.

Those who assigned a score of 1 or 2 were asked to suggest changes, in order to achieve greater clarity and relevance. The Content Validity Index was calculated; and, for items that did not reach the established minimum value (0.8)^(14-15)^, a qualitative step was initiated. At this stage, the suggestions made by the experts were analyzed and incorporated into the instrument.

In addition to the items, each participant also indicated their agreement with the organization of the response scale for each item in ascending order regarding the demand for nursing care. For this assessment, a dichotomous scale (yes or no) was used, and assessments with agreement greater than 70% were considered valid.

In phase 3 of the study, assessment of measurement properties, the instrument resulting from phase 2 was applied over a period of one month to all patients who were admitted to a general adult ward; and the application was made by the nurses themselves, who were already classifying patients in their daily management practice. All nurses who participated in the collection were previously trained by the researchers and were able to clarify their doubts with the author of the instruments.

### Inclusion and exclusion criteria

In phase 2, instrument content validation, seven specialists were selected for convenience: two professors/researchers who worked on the topic of instrument validation, three nurses from nursing management and two nurses from patient care^(15)^. The experience time of these specialists ranged from 7 to 21 years. As an exclusion criterion, participants who did not return the assessment of the instrument’s content within the agreed period were considered.

For phase 3 (assessment of measurement properties), the adapted instrument was applied to all patients hospitalized in a general adult ward, for one month, by the unit’s nurses. The sample size was calculated based on the reference for carrying out factor analysis^(16-18)^, that is, ten patients per item of the instrument. Thus, the minimum sample size calculation resulted in 110 patients. Incomplete classifications were excluded.

### Analysis of results and statistics

Data were tabulated in an Excel for Windows^®^ spreadsheet and analyzed by a statistician using Statistical Analysis Software^®^ (version 9.4) and Partial Least Squares^®^ (PLS, 3.2.1) software.

In phase 2, the CVI for each item was calculated using the following formula^(15)^:


$$CVI=\frac{\sum \text{ number of responses " }3\text{ " or " }4"}{\text{ Total number of responses }}$$

For phase 3, considering that the ICPA was adapted from an instrument with a structure previously defined by exploratory factor analysis^(13)^, it was decided, in the present study, to assess the structural validity, through confirmatory factor analysis. For this, the structural equation model was used, using the PLS as the estimation method.

For this, the convergent validity of the model was first evaluated, through the Average Variance Extracted (AVE) for each of the three domains of the instrument. This measure assesses the proportion of the items’ variance that is explained by the factor to which they belong. AVE values greater than 0.5 indicate that the model converges to a satisfactory result^(16-18)^.

Subsequently, the factor loadings of each indicator within its respective domain were evaluated, with loadings greater than 0.5 being desirable^(16-18)^. The model’s discriminant validity was initially evaluated using the Fornell-Larcker criterion^(16-18)^. This method compares whether the square root of the AVE for a given domain is higher than the correlation values between the domains^(16-18)^. Another criterion considered to assess discriminant validity was the analysis of cross loadings. In this case, it was observed whether the factor loading of a given indicator was higher in the domain in which it was initially allocated^(16-18)^.

In these analyses, it is also possible to assess reliability through the instrument’s internal consistency. Values equal to or greater than 0.6 indicate satisfactory consistency^(16)^.

## RESULTS

In phase 1 of the study, adaptation of the instrument, the PPCI content was adapted to the ICPA, but preserved the same structure, that is, 11 indicators distributed in three domains: “Family” (two indicators), “Patient” (six indicators) and “Therapeutic procedures” (three indicators).

The response scale for each indicator was constructed with four response options (1 to 4 points), and the higher the score, the greater the demand for nursing care. To determine the category of care to which the patient belongs, the nurse must add the points obtained in each of the 11 indicators and classify the patient into minimal (11-17 points), intermediate (18-23 points), high dependency care. (24-30 points), semi-intensive (31-36 points) or intensive (37-44 points).

In phase 2, content validation, only one round was needed for the indicators and the response scale to reach the values established for the CVI and agreement, respectively. These data are presented in Table 1.

**Table 1:** Content Validity Index of the Adult Patient Classification Instrument, Brazil, 2017

Indicator	Content Validity Index	Percentage of agreement with the situations graded on the response scale
Companion participation	0.85	71.4%
Support and support network	1.00	100%
Mental state and activity	1.00	100%
Oxygenation	0.85	85.7%
Mobility and ambulation	1.00	100%
Food and hydration	1.00	71.4%
Eliminations	1.00	71.4%
Hygiene and body care	1.00	100%
Control measurement Interval	1.00	100%
Drug therapy	1.00	85.7%
Cutaneous-mucous integrity	1.00	100%

In phase 3 of the research, the APCI, with its content validated, was applied to 902 patients, however 121 classifications were excluded due to missing data. Thus, 781 classified patients were included in the sample. Among these patients, 39 (5%) were categorized as minimal care; 133 (17%), intermediate care; 375 (48%) of high dependency; 187 (24%), semi-intensive; and 47 (6%), intensive.

**Figure 1:**
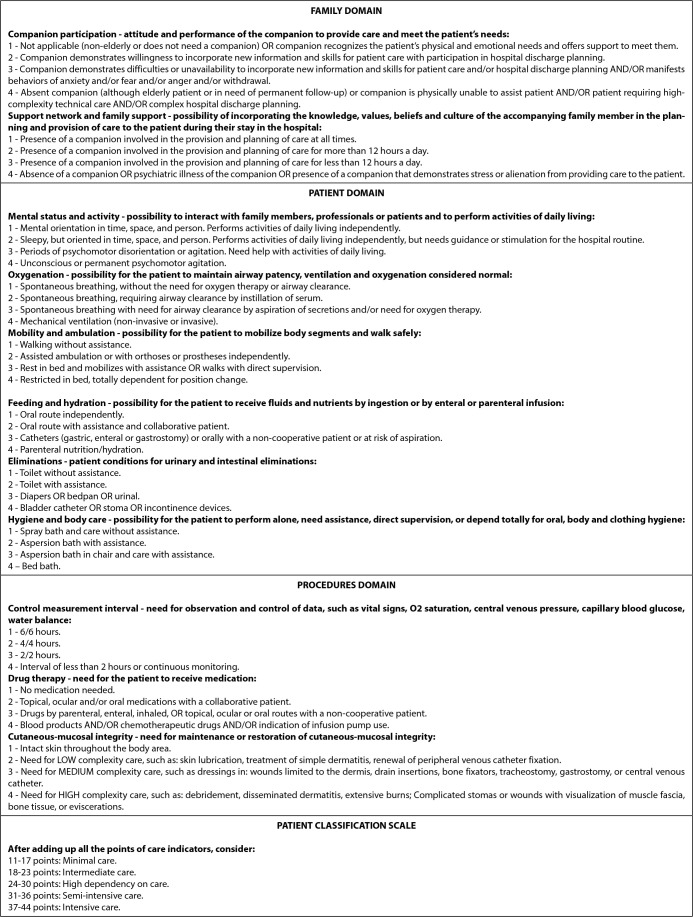
Adult Patient Classification Instrument, Brazil, 2022

The adapted and validated version of the Adult Patient Classification Instrument can be seen in Figure 1.

For the factor analysis, the CVAs and the internal consistency of each of the domains were initially analyzed (Table 2).

**Table 2:** Extracted mean variance, composite reliability, and Cronbach’s alpha of the Adult Patient Classification Instrument dimensions (n = 781), Campinas, São Paulo, Brazil, 2018

Dimensions of the Adult Patient Classification Instrument	Average variance extracted	Composite reliability	Cronbach's alpha
Family	0.94	0.97	0.94
Patient	0.57	0.89	0.84
Procedures	0.52	0.76	0.52

The factor loadings of the items in their respective dimensions and cross factor loadings were presented in Table 3.

**Table 3:** Factor loadings of the indicators in their respective constructs (highlighted) and cross factor loadings (n = 781), Campinas, São Paulo, Brazil, 2018

Indicators	Family	Patient	Procedures
Mental state and activity	-0.02	0.63	0.27
Oxygenation	0.10	0.55	0.41
Mobility and ambulation	0.15	0.88	0.41
Food and hydration	0.06	0.74	0.43
Eliminations	0.16	0.82	0.43
Hygiene and body care	0.19	0.87	0.43
Control measurement interval	0.05	0.47	0.82
Drug therapy	0.03	0.29	0.74
Cutaneous-mucous integrity	0.15	0.35	0.58
Companion participation	0.97	0.14	0.09
Support and support network	0.97	0.15	0.11

The square root of the CVA and the correlations between the constructs were presented in Table 4.

**Table 4:** Square root of the extracted mean variance and correlations between the domains of the Adult Patient Classification Instrument (n = 781), Campinas, São Paulo, Brazil, 2018

Domains	Family	Patient	Procedures
Family	0.97		
Patient	0.15	0.76	
Procedures	0.10	0.52	0.72

## DISCUSSION

Providing an instrument for classifying adult patients that includes the assessment of care demand related to the presence of a family member is extremely important. This is because several publications recommend and/or legitimize the presence of a companion during a patient’s hospital stay, such as the Elderly Statute^(23)^, the SUS humanization policy^(24)^ and the resolution of the National Supplementary Health Agency, which determines that health operators cover the expenses generated by companions^(25)^.

The presence of an instrument that has already been validated in the literature and that considers the family in the demand for care^(13)^ was also essential for carrying out this work, as it converged with recommendations for researchers to adapt instruments to meet new demands, instead of construction of new tools that require more time and resources for their development.

Therefore, the PPCI items were adapted, considering the reality of adult patients, as well as the response options, which were graded in order to respect the dimensionality of previous studies^(13)^.

Content validation is internationally highlighted as the most important property of a tool, as it is at this point that experts judge whether the content is relevant and clear to the target population(15). In this phase, the heterogeneity of experiences of the professionals who composed the sample of specialists was fundamental to provide a relevant, clear, and possible instrument to be used to categorize the care demand of adult patients. In addition, the achievement of an acceptable CVI and agreement, already in the first evaluation round, also demonstrates that the previous phase of the research, adapting the indicators to the reality of adult patients, was carried out with great zeal.

It is interesting to note that, in the indicator “Participation of the companion”, although the quantitative assessments were considered satisfactory (CVI and agreement) by the specialists, some suggestions emerged, such as defining which care could or could not be performed by the companion. This suggestion is very important, since the level of involvement of family members and caregivers in patient care can influence the results and that the aging of the population, as well as the increase in the prevalence of chronic diseases, requires the involvement of the patient and their family with the treatments. proposed^(19-24,26-30)^. However, these suggestions were not incorporated into the instrument, considering that the objective of classifying patients is not to delimit roles in care, but to assess their demand.

It is understood that the presence of a greater number of informal caregivers and the emerging need to formulate policies and rules for the healthy coexistence of caregivers are complementary themes to the analysis of the demand for nursing care and should be considered by the multidisciplinary team to promote greater quality of care. care^(2-3,10,14,26-30)^. In parallel to this, planning for early de-hospitalization and agreement with out-of-hospital caregivers can contribute to reducing early readmissions and, consequently, the costs related to health care^(14,30-37)^.

Regarding the indicators “Food and hydration” and “Eliminations”, an expert suggested that the responses described in alternative 3 (enteral nutrition and use of diapers or bedpans, respectively) be inverted with option 4 (parenteral nutrition and use of bladder catheter or stomas), with the justification that parenteral nutrition and the use of a bladder catheter or stoma required more hours of nursing care when compared to the situations described in option 3. However, this suggestion was not accepted by the authors either, as they considered that The concept of nursing care demand is not restricted to the time spent on direct care activities, as it also includes the complexity of the activities performed, the time for indirect care activities and the severity of the patient’s illness^(37)^.

It is important to highlight that the time of direct care should not be considered superior to the patient’s dependence in relation to care complexity, severity and dependence related to activities of daily living, given that the PCS generically and indirectly capture these load components nursing work^(4)^.

Even linked, it is important to clarify that nursing hours and care demand are not synonymous. Although it is common to associate them, the measurement of nursing time alone reduces care activities similar to an industrial production line, ignoring issues inherent to the art of the profession. The patient’s experience, family dynamics, ethical, social and psychological factors are a multiplicity of caregiving actions and attitudes not restricted to techniques and tasks performed in a specific period of time^(4-6,36)^.

Therefore, it was considered that parenteral nutrition requires care, such as exclusive venous access and good practices for the prevention of bloodstream infection, which make it more complex in relation to enteral nutrition, so enteral nutrition was maintained at 3 points and parenteral rated 4 points. These considerations about the complexity of parenteral nutrition and catheter care were grounded in promoting safe care^(35,37)^.

With regard to the “Eliminations” indicator, the maintenance of ostomies in the 4-point option was based on complex nursing care that ensure the dignity of the patient in the reduction of odors, maintenance of intact skin and delicate techniques for changing stoma bags^(38-39)^ as well as the systematic emptying of the bladder catheter collection bag as a care to prevent infections^(35)^. In addition, it is emphasized that, in the evaluation of this indicator, the inclusion of the caregiver in the preparation for discharge, for daily care and prevention of complications with stomas is also considered^(20,27-35)^.

Following this line of interpretation, the suggestion of a specialist about reversing the situations graded between 3 and 4 points, in the indicator “Drug therapy” was rejected, as it was understood that the non-collaborative patient in the reception of drugs requires less dedication from the nursing care team compared to the administration of chemotherapy, vasoactive drugs, and blood products. This is because the administration of these items transcends the moment of application, as it requires preparation of professionals in pre/post-administration care, such as preparation and installation of therapy and monitoring of vital signs to promote safe and harmless care^(35-36)^.

Confirmatory factor analysis showed the presence of three domains of evaluation of the original instrument^(13)^: “Family” or family support; “Patient” or demand related to specific patient care; and “Therapeutic procedures” or demand related to therapeutic procedures.

The values for CVA, composite reliability, Cronbach’s alpha (with the exception of the therapeutic procedures domain), factor loadings, crossed factor loadings and Fornell-Larcker criterion reached the minimum established by the literature, demonstrating that the model presents satisfactory results, that is, demonstrated evidence of satisfactory validity and reliability^(15-18)^.

Although Cronbach’s alpha of the “Therapeutic Procedures” domain did not reach the established minimum, no modification was made in the domain, as it is understood that the composite reliability is more adequate to the PLS, as it prioritizes the variables according to their reliability, while Cronbach’s alpha is very sensitive to the number of items in each domain^(16)^.

Instruments without evidence of validity and reliability can lead to inadequate diagnosis of problems. Taking into account the enormous applicability of PCI in the management of human and material resources, the methodological robustness of the research that develops these instruments is essential to guarantee a dimensioning of nursing staff and the provision of adequate materials^(1-7,12,31,34)^.

The indicators “Support and family support network” and “Participation of the companion” are the main differentials of the APCI in relation to the other instruments available for the classification of adult patients^(4)^; in addition, it has similarities to instruments validated for neonatal and for binomials in obstetric rooming^(40-41)^.

With the increase in the number of people with chronic diseases, increasingly complex care needs and population aging, the importance of the family member engaged in care in hospital admissions or home care is a reality that cannot be denied^(14,20,35)^. Given the need for instruments that address this dimension, this study came to the understanding that patients, families and/or communities are a unit of care and must be considered for a centered and culturally competent care.

### Study limitations

As a limitation of this study, it can be mentioned the fact that two indicators, suggested by the experts, were not incorporated into the instrument, but which have great potential to influence the demand for care: one related to the use of contact precautions, droplets and/or aerosols; and the other, specifically related to the preparation for hospital discharge. This limitation is intended to be faithful to the structure of the original instrument^(13)^, which does not include these two indicators.

It is understood that the time used to provide care to patients under precaution, intuitively, seems to be longer than that for a patient under standard precaution. However, this information does not necessarily indicate that the patient has greater complexity and/or greater severity, as the patient classification instrument proposes to measure.

Despite this, it is believed that this gap can be filled with further studies and that standard predications can be incorporated into the instrument in the future. Regarding the preparation for discharge, it can be said that this process is the responsibility of the entire multidisciplinary team, so the time required for this practice and the impact on the demand for nursing care also need to be better clarified before this be incorporated into the instrument.

### Contributions to the Area

The availability of an instrument with evidence of reliability and validity that classifies the care demand of adult patients, considering the family assessment, has great potential for clinical and managerial practice. The explanation lies in the fact that this can promote a care environment favorable to the relationship between nurse and family, in order to: build a practice that helps in coping with situations of continued care, since care does not end with hospital discharge; collaborate for a better dimensioning of the nursing team and forecast of material resources.

The APCI can guide the decision-making process in the short term, prioritizing care and relocations that allow the balance of the workforce between shifts^(2-7)^; and in the medium and long term, with the evaluation of the support network and the possibility of family participation in care^(14,20-21,26-32)^, indicators that indirectly help in the planning of hospital discharge.

The systemic assessment of the demand for care, including the assessment of the family dimension, is in agreement with the Global Action Plan for Patient Safety 2021-2030^(35)^. The World Health Organization considers that engaging family members and patients in safe care is a human right, and they should be involved at all levels of care, including sharing decisions. Patients, along with family members or caregivers, have a genuine interest in patient care, so their involvement can make essential contributions to safe care.

## CONCLUSIONS

The present study adapted and made available an instrument for classifying adult patients, considering the family support network in the demand for nursing care. In addition, it tested its measurement properties and demonstrated that the new instrument, called the Adult Patient Classification Instrument, has evidence of validity and reliability.

Therefore, it is recommended to use the instrument in the clinical and managerial practice of nurses to guide decision-making and implementation of strategies that ensure the quality of care offered to the patient and provide support for the management of organizational resources.

## Data Availability

https://doi.org/10.25824/redu/PGYTV5
