# Supplementary material for: Adaptation and validation of an adult patient classification instrument with emphasis on the family dimension
Source: Rev Bras Enferm. 2023 Mar 27;76(2):e20220530. doi: 10.1590/0034-7167-2022-0530 (PMC10042477; doi:10.1590/0034-7167-2022-0530)
Supplement: Supplementary file 1 [file 0034-7167-reben-76-02-e20220530-suppl01.pdf]

Leito/Indicador	Participação do acomp.	Rede de apoio e suporte	Estado mental	Oxigenação	Mobilidade
648A	2	2	3	1	3
648B	2	1	4	3	4
650A	1	1	1	1	2
650B	2	2	4	1	4
652A	1	1	1	1	1
648A	2	1	1	1	3
654A	2	2	3	3	4
654B	1	1	1	1	1
656A	4	4	1	1	2
656B	4	4	1	1	3
658A	1	1	1	1	1
658B	4	4	2	3	3
660A	1	1	1	1	1
660B	2	3	1	1	3
662A	1	1	1	1	1
662B	2	1	1	3	1
662C	4	4	1	3	3
662D	2	1	1	1	2
662C	2	1	1	1	2
658B	1	1	2	3	3
562A	1	3	1	1	1
550B	4	4	2	1	4
560B	1	1	1	1	1
558A	1	2	1	3	2
648A	1	2	1	1	2
648B	1	1	4	3	4
632A	1	1	1	1	1
632D	1	1	1	1	2
634A	1	1	1	1	1
634B	1	1	1	1	4
636A	1	1	1	1	1
638A	1	1	4	4	4
640A	1	1	4	4	4
642A	1	1	1	1	1
642B	1	1	1	1	2
644A	4	3	2	1	2
646A	1	1	1	1	1
646B	1	1	1	1	1
650B	1	1	1	1	3
656A	4	4	1	3	3
656B	2	1	1	4	3
658A	1	1	1	1	1
648A	1	1	1	1	1
648B	4	4	2	1	3
654A	4	4	1	1	3
654B	1	1	1	1	2
649A	2	1	1	1	2
649B	2	1	1	1	1

651A	1	1	2	3	3
651B	1	1	1	1	3
653A	2	2	1	1	3
653B	4	1	1	3	3
655A	4	4	3	3	4
657A	2	1	1	1	3
657B	1	1	1	1	2
659A	1	1	1	1	2
659B	1	1	1	1	2
661B	1	1	1	1	3
663A	1	1	1	1	1
663B	1	1	1	1	1
663C	1	1	1	3	2
663D	1	2	1	1	2
550B	4	4	1	1	4
552A	4	4	1	3	2
552B	3	4	1	3	4
554A	4	4	1	1	4
554B	1	1	1	1	2
556A	1	1	1	1	1
556B	1	1	1	1	1
558A	2	1	1	3	3
558B	4	4	1	1	3
560A	4	4	2	1	3
560B	1	1	1	1	1
562A	1	1	1	1	1
562C	4	4	1	1	1
562D	1	1	1	1	1
500C	1	1	1	1	2
500D	1	1	1	1	1
502A	4	4	1	1	1
504B	1	1	1	1	1
506A	4	4	2	1	2
506B	1	1	1	1	1
501A	1	1	1	1	3
501C	1	1	1	1	3
503B	4	4	3	1	3
507A	1	1	1	3	3
507B	2	1	1	1	2
513A	1	1	1	1	3
513B	1	1	1	1	2
519B	1	1	1	1	3
527A	1	1	1	1	3
531B	1	1	1	1	2
531C	2	2	1	1	2
648A	4	4	1	1	1
654A	4	4	2	1	3
654B	4	4	1	1	1
658A	1	1	1	1	1
660A	4	4	1	1	1

662C	2	1	3	3	3
662D	1	1	1	1	2
	Domínio Família		Domínio Paciente		

Alimentação	Eliminações	Higiene	Intervalo de aferição	Terapêutica	Integridade cut-muc	Total
2	3	4	4	4	2	**30**
2	4	4	1	3	3	**31**
1	4	2	1	3	3	**20**
1	1	1	1	3	2	**22**
1	1	1	1	3	2	**14**
1	2	2	1	3	4	**21**
2	4	4	1	3	2	**30**
1	1	1	4	4	2	**18**
2	2	2	1	3	2	**24**
1	4	4	1	3	2	**28**
1	1	2	1	3	3	**16**
2	3	3	1	3	2	**30**
1	1	1	1	3	3	**15**
2	4	3	1	3	2	**25**
1	1	1	1	3	2	**14**
1	1	1	1	3	2	**17**
2	4	4	1	3	2	**31**
1	3	2	1	3	3	**20**
2	3	3	1	3	2	**21**
2	3	3	4	3	2	**27**
1	1	1	1	3	2	**16**
3	4	4	2	3	3	**34**
1	1	1	1	2	1	**12**
1	2	3	1	3	3	**22**
1	3	3	1	3	4	**22**
2	4	4	4	3	4	**34**
1	1	1	1	2	3	**14**
1	1	1	1	2	3	**15**
1	1	1	1	1	1	**11**
2	4	4	4	3	1	**26**
1	1	1	1	1	1	**11**
4	4	4	4	4	2	**36**
4	4	4	4	4	4	**38**
1	1	2	1	2	1	**13**
1	1	2	1	2	1	**14**
2	2	3	1	2	1	**23**
1	1	1	1	2	1	**12**
1	1	1	1	3	1	**13**
1	2	3	1	3	3	**20**
1	2	3	1	3	2	**27**
1	3	3	1	3	2	**24**
1	1	1	1	3	2	**14**
1	1	1	1	3	2	**14**
1	3	4	1	3	3	**29**
1	3	3	1	3	3	**27**
1	1	1	1	3	2	**15**
1	2	2	1	2	2	**17**
1	1	1	1	1	4	**15**

2	3	4	1	3	2	**25**
2	3	4	1	3	2	**22**
1	3	3	1	3	1	**21**
1	3	4	1	3	1	**25**
3	4	4	2	3	4	**38**
2	3	3	1	3	4	**24**
1	3	2	1	3	1	**17**
1	2	2	1	3	1	**16**
1	2	2	1	3	1	**16**
1	3	2	1	3	4	**21**
1	1	1	1	1	1	**11**
1	1	1	1	3	2	**14**
1	4	3	4	3	3	**26**
1	1	2	1	3	1	**16**
3	3	4	1	3	3	**31**
1	2	2	1	3	3	**26**
2	4	4	1	3	2	**31**
1	3	4	4	3	3	**32**
1	3	3	1	3	3	**20**
1	1	1	1	3	2	**14**
1	1	1	1	4	1	**14**
1	3	2	1	3	3	**23**
3	3	4	1	3	3	**30**
3	2	3	1	3	2	**28**
2	1	1	1	3	2	**15**
1	1	2	1	3	2	**15**
1	2	2	1	3	1	**21**
1	1	1	1	3	2	**14**
1	2	1	1	3	2	**16**
1	1	1	1	1	1	**11**
2	1	1	1	3	3	**22**
2	1	1	1	3	2	**15**
3	3	4	4	4	3	**34**
1	1	1	1	3	2	**14**
1	2	3	1	3	4	**21**
1	2	2	1	3	4	**20**
1	3	4	1	3	3	**30**
1	3	4	1	3	3	**24**
1	2	3	1	3	4	**21**
1	2	2	1	4	3	**20**
1	3	2	1	3	3	**19**
2	4	3	1	3	3	**23**
1	2	2	1	3	3	**19**
1	3	3	1	3	3	**20**
1	2	2	1	3	4	**21**
1	4	2	1	3	2	**24**
2	3	3	1	3	2	**28**
1	1	1	1	3	2	**20**
1	1	1	1	2	2	**13**
1	1	1	1	3	2	**20**

2	3	3	1	3	2	**26**
2	1	1	1	3	4	**18**

Domínio Procedimentos Terapêuticos

Classificação	Data e local coleta
Alta dep.	urgesp 2/05
Semi-int.	urgesp 2/05
intermed.	urgesp 2/05
intermed.	urgesp 3/05
mínimos	urgesp 3/05
intermed.	urgesp 3/05
alta dep.	urgesp 3/05
intermed.	urgesp 3/05
alta-dep.	urgesp 3/05
alta-dep.	urgesp 3/05
mínimos	urgesp 3/05
alta-dep.	urgesp 3/05
mínimos	urgesp 3/05
alta-dep.	urgesp 3/05
mínimos	urgesp 3/05
mínimos	urgesp 3/05
semi-int.	urgesp 3/05
intermed.	urgesp 3/05
intermed.	urgesp 4/05
alta-dep.	urgesp 4/05
mínimos	cardio 4/05
semi-int.	cardio 4/05
mínimos	cardio 4/05
intermed.	cardio 4/05
intermed.	urgesp 4/05
semi-int.	urgesp 4/05
mínimos	MI 5/05
mínimos	MI 5/05
mínimos	MI 5/05
alta-dep.	MI 5/05
mínimos	MI 5/05
semi-int.	MI 5/05
intensivo	MI 5/05
mínimos	MI 5/05
mínimos	MI 5/05
intermed.	MI 5/05
mínimos	MI 5/05
mínimos	MI 5/05
intermed.	urgesp 5/05
alta-dep.	urgesp 5/05
alta-dep.	urgesp 5/05
mínimos	urgesp 5/05
mínimos	urgesp 8/05
alta-dep.	urgesp 8/05
alta-dep.	urgesp 8/05
mínimos	urgesp 8/05
mínimos	EGA 8/05
mínimos	EGA 8/05

alta-dep.	EGA 8/05
intermed.	EGA 8/05
intermed.	EGA 8/05
alta-dep.	EGA 8/05
intensivo	EGA 8/05
alta-dep.	EGA 8/05
mínimos	EGA 8/05
mínimos	EGA 8/05
mínimos	EGA 8/05
intermed.	EGA 8/05
mínimos	EGA 8/05
mínimos	EGA 8/05
alta-dep.	EGA 8/05
mínimos	EGA 8/05
semi-int	cardio 10/5
alta-dep	cardio 10/5
semi-int.	cardio 10/5
semi-int.	cardio 10/5
intermed.	cardio 10/5
mínimos	cardio 10/5
mínimos	cardio 10/5
intermed.	cardio 10/5
alta-dep.	cardio 10/5
alta-dep.	cardio 10/5
mínimos	cardio 10/5
mínimos	cardio 10/5
intermed.	cardio 10/5
mínimos	cardio 10/5
mínimos	neuro 10/5
mínimos	neuro 10/5
intermed.	neuro 10/5
mínimos	neuro 10/5
semi-int.	neuro 10/5
mínimos	neuro 10/5
intermed.	ortop 10/5
intermed.	ortop 10/5
alta-dep.	ortop 10/5
alta-dep.	ortop 10/5
intermed.	ortop 10/5
intermed.	ortop 10/5
intermed.	ortop 10/5
intermed.	trauma 10/5
intermed.	trauma 10/5
intermed.	trauma 10/5
intermed.	trauma 10/5
alta-dep.	urgesp 11/5
alta-dep.	urgesp 11/5
intermed.	urgesp 11/5
mínimos	urgesp 11/5
intermed.	urgesp 11/5

alta-dep.	urgesp 11/5
intermed.	urgesp 11/5

Indicador/Leito	648A	648B	650A	650B	652A	648A	654A
Participação do acomp.	2	2	1	2	1	2	2
Rede de apoio e suporte	2	1	1	2	1	1	2
Estado mental	3	4	1	4	1	1	3
Oxigenação	1	3	1	1	1	1	3
Mobilidade	3	4	2	4	1	3	4
Alimentação	2	2	1	1	1	1	2
Eliminações	3	4	4	1	1	2	4
Higiene	4	4	2	1	1	2	4
Intervalo de aferição	4	1	1	1	1	1	1
Terapêutica	4	3	3	3	3	3	3
Integridade	2	3	3	2	2	4	2
**Total**	**30**	**31**	**20**	**22**	**14**	**21**	**30**
**Classificação**	Alta dep.	Semi-int.	intermed.	intermed.	mínimos	intermed.	alta dep.
	urgesp 2/05	urgesp 2/05	urgesp 2/05	urgesp 3/05	urgesp 3/05	urgesp 3/05	urgesp 3/05

654B	656A	656B	658A	658B	660A	660B	662A	662B	662C
1	4	4	1	4	1	2	1	2	4
1	4	4	1	4	1	3	1	1	4
1	1	1	1	2	1	1	1	1	1
1	1	1	1	3	1	1	1	3	3
1	2	3	1	3	1	3	1	1	3
1	2	1	1	2	1	2	1	1	2
1	2	4	1	3	1	4	1	1	4
1	2	4	2	3	1	3	1	1	4
4	1	1	1	1	1	1	1	1	1
4	3	3	3	3	3	3	3	3	3
2	2	2	3	2	3	2	2	2	2
**18**	**24**	**28**	**16**	**30**	**15**	**25**	**14**	**17**	**31**
intermed.	alta-dep.	alta-dep.	mínimos	alta-dep.	mínimos	alta-dep.	mínimos	mínimos	semi-int.
urgesp 3/05	urgesp 3/05	urgesp 3/05	urgesp 3/05	urgesp 3/05	urgesp 3/05	urgesp 3/05	urgesp 3/05	urgesp 3/05	urgesp 3/05

662D	662C	658B	562A	550B	560B	558A	648A	648B	632A
2	2	1	1	4	1	1	1	1	1
1	1	1	3	4	1	2	2	1	1
1	1	2	1	2	1	1	1	4	1
1	1	3	1	1	1	3	1	3	1
2	2	3	1	4	1	2	2	4	1
1	2	2	1	3	1	1	1	2	1
3	3	3	1	4	1	2	3	4	1
2	3	3	1	4	1	3	3	4	1
1	1	4	1	2	1	1	1	4	1
3	3	3	3	3	2	3	3	3	2
3	2	2	2	3	1	3	4	4	3
**20**	**21**	**27**	**16**	**34**	**12**	**22**	**22**	**34**	**14**
intermed.	intermed.	alta-dep.	mínimos	semi-int.	mínimos	intermed.	intermed.	semi-int.	mínimos
urgesp 3/05	urgesp 4/05	urgesp 4/05	cardio 4/05	cardio 4/05	cardio 4/05	cardio 4/05	urgesp 4/05	urgesp 4/05	MI 5/05

632D	634A	634B	636A	638A	640A	642A	642B	644A	646A
1	1	1	1	1	1	1	1	4	1
1	1	1	1	1	1	1	1	3	1
1	1	1	1	4	4	1	1	2	1
1	1	1	1	4	4	1	1	1	1
2	1	4	1	4	4	1	2	2	1
1	1	2	1	4	4	1	1	2	1
1	1	4	1	4	4	1	1	2	1
1	1	4	1	4	4	2	2	3	1
1	1	4	1	4	4	1	1	1	1
2	1	3	1	4	4	2	2	2	2
3	1	1	1	2	4	1	1	1	1
**15**	**11**	**26**	**11**	**36**	**38**	**13**	**14**	**23**	**12**
mínimos	mínimos	alta-dep.	mínimos	semi-int.	intensivo	mínimos	mínimos	intermed.	mínimos
MI 5/05	MI 5/05	MI 5/05	MI 5/05	MI 5/05	MI 5/05	MI 5/05	MI 5/05	MI 5/05	MI 5/05

646B	650B	656A	656B	658A	648A	648B	654A	654B	649A
1	1	4	2	1	1	4	4	1	2
1	1	4	1	1	1	4	4	1	1
1	1	1	1	1	1	2	1	1	1
1	1	3	4	1	1	1	1	1	1
1	3	3	3	1	1	3	3	2	2
1	1	1	1	1	1	1	1	1	1
1	2	2	3	1	1	3	3	1	2
1	3	3	3	1	1	4	3	1	2
1	1	1	1	1	1	1	1	1	1
3	3	3	3	3	3	3	3	3	2
1	3	2	2	2	2	3	3	2	2
**13**	**20**	**27**	**24**	**14**	**14**	**29**	**27**	**15**	**17**
mínimos	intermed.	alta-dep.	alta-dep.	mínimos	mínimos	alta-dep.	alta-dep.	mínimos	mínimos
MI 5/05	urgesp 5/05	urgesp 5/05	urgesp 5/05	urgesp 5/05	urgesp 8/05	urgesp 8/05	urgesp 8/05	urgesp 8/05	EGA 8/05

649B	651A	651B	653A	653B	655A	657A	657B	659A	659B
2	1	1	2	4	4	2	1	1	1
1	1	1	2	1	4	1	1	1	1
1	2	1	1	1	3	1	1	1	1
1	3	1	1	3	3	1	1	1	1
1	3	3	3	3	4	3	2	2	2
1	2	2	1	1	3	2	1	1	1
1	3	3	3	3	4	3	3	2	2
1	4	4	3	4	4	3	2	2	2
1	1	1	1	1	2	1	1	1	1
1	3	3	3	3	3	3	3	3	3
4	2	2	1	1	4	4	1	1	1
**15**	**25**	**22**	**21**	**25**	**38**	**24**	**17**	**16**	**16**
mínimos	alta-dep.	intermed.	intermed.	alta-dep.	intensivo	alta-dep.	mínimos	mínimos	mínimos
EGA 8/05	EGA 8/05	EGA 8/05	EGA 8/05	EGA 8/05	EGA 8/05	EGA 8/05	EGA 8/05	EGA 8/05	EGA 8/05

661B	663A	663B	663C	663D	550B	552A	552B	554A	554B
1	1	1	1	1	4	4	3	4	1
1	1	1	1	2	4	4	4	4	1
1	1	1	1	1	1	1	1	1	1
1	1	1	3	1	1	3	3	1	1
3	1	1	2	2	4	2	4	4	2
1	1	1	1	1	3	1	2	1	1
3	1	1	4	1	3	2	4	3	3
2	1	1	3	2	4	2	4	4	3
1	1	1	4	1	1	1	1	4	1
3	1	3	3	3	3	3	3	3	3
4	1	2	3	1	3	3	2	3	3
**21**	**11**	**14**	**26**	**16**	**31**	**26**	**31**	**32**	**20**
intermed.	mínimos	mínimos	alta-dep.	mínimos	semi-int	alta-dep	semi-int.	semi-int.	intermed.
EGA 8/05	EGA 8/05	EGA 8/05	EGA 8/05	EGA 8/05	cardio 10/5	cardio 10/5	cardio 10/5	cardio 10/5	cardio 10/5

556A	556B	558A	558B	560A	560B	562A	562C	562D	500C
1	1	2	4	4	1	1	4	1	1
1	1	1	4	4	1	1	4	1	1
1	1	1	1	2	1	1	1	1	1
1	1	3	1	1	1	1	1	1	1
1	1	3	3	3	1	1	1	1	2
1	1	1	3	3	2	1	1	1	1
1	1	3	3	2	1	1	2	1	2
1	1	2	4	3	1	2	2	1	1
1	1	1	1	1	1	1	1	1	1
3	4	3	3	3	3	3	3	3	3
2	1	3	3	2	2	2	1	2	2
**14**	**14**	**23**	**30**	**28**	**15**	**15**	**21**	**14**	**16**
mínimos	mínimos	intermed.	alta-dep.	alta-dep.	mínimos	mínimos	intermed.	mínimos	mínimos
cardio 10/5	cardio 10/5	cardio 10/5	cardio 10/5	cardio 10/5	cardio 10/5	cardio 10/5	cardio 10/5	cardio 10/5	neuro 10/5

500D	502A	504B	506A	506B	501A	501C	503B	507A	507B
1	4	1	4	1	1	1	4	1	2
1	4	1	4	1	1	1	4	1	1
1	1	1	2	1	1	1	3	1	1
1	1	1	1	1	1	1	1	3	1
1	1	1	2	1	3	3	3	3	2
1	2	2	3	1	1	1	1	1	1
1	1	1	3	1	2	2	3	3	2
1	1	1	4	1	3	2	4	4	3
1	1	1	4	1	1	1	1	1	1
1	3	3	4	3	3	3	3	3	3
1	3	2	3	2	4	4	3	3	4
**11**	**22**	**15**	**34**	**14**	**21**	**20**	**30**	**24**	**21**
mínimos	intermed.	mínimos	semi-int.	mínimos	intermed.	intermed.	alta-dep.	alta-dep.	intermed.
neuro 10/5	neuro 10/5	neuro 10/5	neuro 10/5	neuro 10/5	ortop 10/5	ortop 10/5	ortop 10/5	ortop 10/5	ortop 10/5

513A	513B	519B	527A	531B	531C	648A	654A	654B	658A
1	1	1	1	1	2	4	4	4	1
1	1	1	1	1	2	4	4	4	1
1	1	1	1	1	1	1	2	1	1
1	1	1	1	1	1	1	1	1	1
3	2	3	3	2	2	1	3	1	1
1	1	2	1	1	1	1	2	1	1
2	3	4	2	3	2	4	3	1	1
2	2	3	2	3	2	2	3	1	1
1	1	1	1	1	1	1	1	1	1
4	3	3	3	3	3	3	3	3	2
3	3	3	3	3	4	2	2	2	2
**20**	**19**	**23**	**19**	**20**	**21**	**24**	**28**	**20**	**13**
intermed.	intermed.	intermed.	intermed.	intermed.	intermed.	alta-dep.	alta-dep.	intermed.	mínimos
ortop 10/5	ortop 10/5	trauma 10/5	trauma 10/5	trauma 10/5	trauma 10/5	urgesp 11/5	urgesp 11/5	urgesp 11/5	urgesp 11/5

660A	662C	662D
4	2	1
4	1	1
1	3	1
1	3	1
1	3	2
1	2	2
1	3	1
1	3	1
1	1	1
3	3	3
2	2	4
**20**	**26**	**18**
intermed.	alta-dep.	intermed.
urgesp 11/5	urgesp 11/5	urgesp 11/5

cardio 10/5
cardio 10/5
cardio 10/5
cardio 10/5
cardio 10/5
cardio 10/5
cardio 10/5
cardio 10/5
cardio 10/5
cardio 10/5
cardio 10/5
cardio 10/5
cardio 10/5
cardio 10/5
cardio 4/05
cardio 4/05
cardio 4/05
cardio 4/05
EGA 8/05
EGA 8/05
EGA 8/05
EGA 8/05
EGA 8/05
EGA 8/05
EGA 8/05
EGA 8/05
EGA 8/05
EGA 8/05
EGA 8/05
EGA 8/05
EGA 8/05
EGA 8/05
EGA 8/05
EGA 8/05
MI 5/05
MI 5/05
MI 5/05
MI 5/05
MI 5/05
MI 5/05
MI 5/05
MI 5/05
MI 5/05
MI 5/05
MI 5/05
MI 5/05
neuro 10/5

neuro 10/5
neuro 10/5
neuro 10/5
neuro 10/5
neuro 10/5
ortop 10/5
ortop 10/5
ortop 10/5
ortop 10/5
ortop 10/5
ortop 10/5
ortop 10/5
trauma 10/5
trauma 10/5
trauma 10/5
trauma 10/5
urgesp 11/5
urgesp 11/5
urgesp 11/5
urgesp 11/5
urgesp 11/5
urgesp 11/5
urgesp 11/5
urgesp 2/05
urgesp 2/05
urgesp 2/05
urgesp 3/05
urgesp 3/05
urgesp 3/05
urgesp 3/05
urgesp 3/05
urgesp 3/05
urgesp 3/05
urgesp 3/05
urgesp 3/05
urgesp 3/05
urgesp 3/05
urgesp 3/05
urgesp 3/05
urgesp 3/05
urgesp 3/05
urgesp 4/05
urgesp 4/05
urgesp 4/05
urgesp 4/05
urgesp 5/05
urgesp 5/05

urgesp 5/05
urgesp 5/05
urgesp 8/05
urgesp 8/05
urgesp 8/05
urgesp 8/05
