# Supplementary material for: Adaptation and validation of an adult patient classification instrument with emphasis on the family dimension
Source: Rev Bras Enferm. 2023 Mar 27;76(2):e20220530. doi: 10.1590/0034-7167-2022-0530 (PMC10042477; doi:10.1590/0034-7167-2022-0530)
Supplement: Supplementary file 2 [file 0034-7167-reben-76-02-e20220530-suppl02.pdf]

# ENFERMARIA GERAL DE ADULTOS/ CLASSIFICAÇÃO DE PACIENTES/ INSTRUMENTO DE DINI ADAPTADO PARA ADULTOS

| | Pontuação/Leito | 633A | 633B | 633C | 633D | 635A | 635B | 637A | 637B | 639A | 639B | 641A | 641B | 643A | 643B | 645A | 645B | 647A | 647B |
|---|---|---|---|---|---|---|---|---|---|---|---|---|---|---|---|---|---|---|---|
| Estado Mental e Atividade | 1 - Orientação mental em tempo, espaço e pessoa. Realiza atividades de vida diária com independência. | | | | | | | | | | | | | | | | | | |
| | 2 - Sonolento, mas orientado em tempo, espaço e pessoa. Realiza atividades de vida diária com independência, mas necessita de orientação ou estímulo para a rotina hospitalar. | | | | | | | | | | | | | | | | | | |
| | 3 - Períodos de desorientação ou agitação psicomotora. Necessita de auxílio para atividades de vida diária. | | | | | | | | | | | | | | | | | | |
| | 4 - Inconsciente OU agitação psicomotora permanente. | | | | | | | | | | | | | | | | | | |
| Oxigenação | 1 - Respiração espontânea, sem necessidade de oxigenoterapia ou de desobstrução de vias aéreas. | | | | | | | | | | | | | | | | | | |
| | 2 - Respiração espontânea com necessidade de desobstrução de vias aéreas por instilação de soro. | | | | | | | | | | | | | | | | | | |
| | 3 - Respiração espontânea com necessidade de desobstrução de vias aéreas por aspiração de secreções E/OU necessidade de oxigenoterapia. | | | | | | | | | | | | | | | | | | |
| | 4 - Ventilação mecânica (não invasiva ou invasiva). | | | | | | | | | | | | | | | | | | |
| Mobilidade e Deambulação | 1 - Deambulação sem auxílio. | | | | | | | | | | | | | | | | | | |
| | 2 - Deambulação assistida ou com órteses. | | | | | | | | | | | | | | | | | | |
| | 3 - Repouso no leito e mobilização com auxílio OU deambula com supervisão direta. | | | | | | | | | | | | | | | | | | |
| | 4 - Restrito no leito, totalmente dependente para mudança de decúbito. | | | | | | | | | | | | | | | | | | |
| Alimentação e Hidratação | 1 - Via oral de forma independente. | | | | | | | | | | | | | | | | | | |
| | 2 - Via oral com auxílio e paciente colaborativo. | | | | | | | | | | | | | | | | | | |
| | 3 - Sondas (gástrica, enteral ou gastrostomia) OU via oral com paciente não colaborativo ou com risco de aspiração. | | | | | | | | | | | | | | | | | | |
| | 4 - Nutrição/ Hidratação parenteral. | | | | | | | | | | | | | | | | | | |
| Eliminações | 1 - Vaso sanitário sem auxílio. | | | | | | | | | | | | | | | | | | |
| | 2 - Vaso sanitário com auxílio. | | | | | | | | | | | | | | | | | | |
| | 3 - Fraldas OU comadre OU urinol OU uropen. | | | | | | | | | | | | | | | | | | |
| | 4 - Sonda vesical OU estomas. | | | | | | | | | | | | | | | | | | |

| | Pontuação/Leito | 633A | 633B | 633C | 633D | 635A | 635B | 637A | 637B | 639A | 639B | 641A | 641B | 643A | 643B | 645A | 645B | 647A | 647B |
|---|---|---|---|---|---|---|---|---|---|---|---|---|---|---|---|---|---|---|---|
| Higiene e Cuidado Corporal | 1 - Banho de aspersão e cuidados sem auxílio. | | | | | | | | | | | | | | | | | | |
| | 2 - Banho de aspersão com auxílio parcial. | | | | | | | | | | | | | | | | | | |
| | 3 - Banho de aspersão em cadeira ou com auxílio total. | | | | | | | | | | | | | | | | | | |
| | 4 - Banho no leito. | | | | | | | | | | | | | | | | | | |
| Intervalo de Aferição de Controles | 1 - 6/6 horas. | | | | | | | | | | | | | | | | | | |
| | 2 - 4/4 horas. | | | | | | | | | | | | | | | | | | |
| | 3 - 2/2 horas. | | | | | | | | | | | | | | | | | | |
| | 4 - Intervalo menor de 2 horas OU monitorização contínua. | | | | | | | | | | | | | | | | | | |
| Terapêutica Medicamentosa | 1 - Não necessita de medicamentos. | | | | | | | | | | | | | | | | | | |
| | 2 - Medicamentos por via tópica, ocular E/OU oral com paciente colaborativo. | | | | | | | | | | | | | | | | | | |
| | 3 - Medicamentos por vias parenteral, enteral, inalatória; OU por via tópica, ocular ou oral com paciente não colaborativo. | | | | | | | | | | | | | | | | | | |
| | 4 - Hemoderivados E/OU quimioterápicos E/OU indicação absoluta de uso de bomba de infusão. | | | | | | | | | | | | | | | | | | |
| Integridade Cutâneo Mucosa | 1 - Pele íntegra em toda a área corpórea. | | | | | | | | | | | | | | | | | | |
| | 2 - Necessidade de cuidados de BAIXA complexidade como: lubrificação da pele, tratamento de dermatites simples, renovação de fixação de cateter venoso periférico. | | | | | | | | | | | | | | | | | | |
| | 3 - Necessidade de cuidados de MÉDIA complexidade como curativos em: feridas limitadas à derme, inserções de drenos, traqueostomia, gastrostomia ou de cateter venoso central. | | | | | | | | | | | | | | | | | | |
| | 4 - Necessidade de cuidados de ALTA complexidade como: desbridamentos, dermatites disseminadas, queimaduras extensas, estomas complexos ou feridas com visualização de fáscia muscular, tecido ósseo ou eviscerações. | | | | | | | | | | | | | | | | | | |

| | Pontuação/Leito | 633A | 633B | 633C | 633D | 635A | 635B | 637A | 637B | 639A | 639B | 641A | 641B | 643A | 643B | 645A | 645B | 647A | 647B |
|---|---|---|---|---|---|---|---|---|---|---|---|---|---|---|---|---|---|---|---|
| **Participação do acompanhante** | 1 - Não se aplica (não idoso ou não precisa de acompanhante) OU acompanhante reconhece as necessidades físicas e emocionais do paciente e oferece suporte para atendê-las. | | | | | | | | | | | | | | | | | | |
| | 2 - Acompanhante demonstra disponibilidade para incorporar novas informações e habilidades para o cuidado do paciente com participação no planejamento de alta hospitalar. | | | | | | | | | | | | | | | | | | |
| | 3 - Acompanhante demonstra dificuldades ou indisponibilidade para incorporar novas informações e habilidades para o cuidado do paciente E/OU manifesta comportamentos de ansiedade E/OU medo E/OU raiva E/OU retraimento. | | | | | | | | | | | | | | | | | | |
| | 4 - Acompanhante ausente (embora paciente idoso ou com necessidades de acompanhamento permanente) OU acompanhante com impossibilidade física de auxiliar paciente E/OU paciente requer cuidados técnicos de alta complexidade. | | | | | | | | | | | | | | | | | | |
| **Rede de apoio e suporte** | 1 - Presença de um acompanhante envolvido na prestação e planejamento de cuidados durante todo o tempo OU não se aplica (não idoso ou não precisa de acompanhante). | | | | | | | | | | | | | | | | | | |
| | 2 - Presença de um acompanhante envolvido na prestação e planejamento de cuidados durante mais de 12 horas ao dia. | | | | | | | | | | | | | | | | | | |
| | 3 - Presença de acompanhante envolvido na prestação e planejamento de cuidados durante menos de 12 horas ao dia. | | | | | | | | | | | | | | | | | | |
| | 4 - Ausência de acompanhante OU doença psiquiátrica do acompanhante OU presença de acompanhante que demonstre estresse ou alienação da prestação de cuidados ao paciente. | | | | | | | | | | | | | | | | | | |

**CLASSIFICAÇÃO:** Escore total _____ pontos. **CONSIDERAR:** Cuidados mínimos (11-17 pontos); Cuidados intermediários (18-23 pontos); Alta dependência (24-30 pontos); Cuidados semi intensivos (31-36 pontos); Cuidados intensivos (37-44 pontos).

# ENFERMARIA GERAL DE ADULTOS/ CLASSIFICAÇÃO DE PACIENTES/ INSTRUMENTO DE DINI ADAPTADO PARA ADULTOS

| | Pontuação/Leito | 649A | 649B | 651A | 651B | 653A | 653B | 655A | 655B | 657A | 657B | 659A | 659B | 661A | 661B | 663A | 663B | 663C | 663D |
|---|---|---|---|---|---|---|---|---|---|---|---|---|---|---|---|---|---|---|---|
| Estado Mental e Atividade | 1 - Orientação mental em tempo, espaço e pessoa. Realiza atividades de vida diária com independência. | | | | | | | | | | | | | | | | | | |
| | 2 - Sonolento, mas orientado em tempo, espaço e pessoa. Realiza atividades de vida diária com independência, mas necessita de orientação ou estímulo para a rotina hospitalar. | | | | | | | | | | | | | | | | | | |
| | 3 - Períodos de desorientação ou agitação psicomotora. Necessita de auxílio para atividades de vida diária. | | | | | | | | | | | | | | | | | | |
| | 4 - Inconsciente OU agitação psicomotora permanente. | | | | | | | | | | | | | | | | | | |
| Oxigenação | 1 - Respiração espontânea, sem necessidade de oxigenoterapia ou de desobstrução de vias aéreas. | | | | | | | | | | | | | | | | | | |
| | 2 - Respiração espontânea com necessidade de desobstrução de vias aéreas por instilação de soro. | | | | | | | | | | | | | | | | | | |
| | 3 - Respiração espontânea com necessidade de desobstrução de vias aéreas por aspiração de secreções E/OU necessidade de oxigenoterapia. | | | | | | | | | | | | | | | | | | |
| | 4 - Ventilação mecânica (não invasiva ou invasiva). | | | | | | | | | | | | | | | | | | |
| Mobilidade e Deambulação | 1 - Deambulação sem auxílio. | | | | | | | | | | | | | | | | | | |
| | 2 - Deambulação assistida ou com órteses. | | | | | | | | | | | | | | | | | | |
| | 3 - Repouso no leito e mobilização com auxílio OU deambula com supervisão direta. | | | | | | | | | | | | | | | | | | |
| | 4 - Restrito no leito, totalmente dependente para mudança de decúbito. | | | | | | | | | | | | | | | | | | |
| Alimentação e Hidratação | 1 - Via oral de forma independente. | | | | | | | | | | | | | | | | | | |
| | 2 - Via oral com auxílio e paciente colaborativo. | | | | | | | | | | | | | | | | | | |
| | 3 - Sondas (gástrica, enteral ou gastrostomia) OU via oral com paciente não colaborativo ou com risco de aspiração. | | | | | | | | | | | | | | | | | | |
| | 4 - Nutrição/ Hidratação parenteral. | | | | | | | | | | | | | | | | | | |
| Eliminações | 1 - Vaso sanitário sem auxílio. | | | | | | | | | | | | | | | | | | |
| | 2 - Vaso sanitário com auxílio. | | | | | | | | | | | | | | | | | | |
| | 3 - Fraldas OU comadre OU urinol OU uropen. | | | | | | | | | | | | | | | | | | |
| | 4 - Sonda vesical OU estomas. | | | | | | | | | | | | | | | | | | |

| | Pontuação/Leito | 649A | 649B | 651A | 651B | 653A | 653B | 655A | 655B | 657A | 657B | 659A | 659B | 661A | 661B | 663A | 663B | 663C | 663D |
|---|---|---|---|---|---|---|---|---|---|---|---|---|---|---|---|---|---|---|---|
| Higiene e Cuidado Corporal | 1 - Banho de aspersão e cuidados sem auxílio. | | | | | | | | | | | | | | | | | | |
| | 2 - Banho de aspersão com auxílio parcial. | | | | | | | | | | | | | | | | | | |
| | 3 - Banho de aspersão em cadeira ou com auxílio total. | | | | | | | | | | | | | | | | | | |
| | 4 - Banho no leito. | | | | | | | | | | | | | | | | | | |
| Intervalo de Aferição de Controles | 1 - 6/6 horas. | | | | | | | | | | | | | | | | | | |
| | 2 - 4/4 horas. | | | | | | | | | | | | | | | | | | |
| | 3 - 2/2 horas. | | | | | | | | | | | | | | | | | | |
| | 4 - Intervalo menor de 2 horas OU monitorização contínua. | | | | | | | | | | | | | | | | | | |
| Terapêutica Medicamentosa | 1 - Não necessita de medicamentos. | | | | | | | | | | | | | | | | | | |
| | 2 - Medicamentos por via tópica, ocular E/OU oral com paciente colaborativo. | | | | | | | | | | | | | | | | | | |
| | 3 - Medicamentos por vias parenteral, enteral, inalatória; OU por via tópica, ocular ou oral com paciente não colaborativo. | | | | | | | | | | | | | | | | | | |
| | 4 - Hemoderivados E/OU quimioterápicos E/OU indicação absoluta de uso de bomba de infusão. | | | | | | | | | | | | | | | | | | |
| Integridade Cutâneo Mucosa | 1 - Pele íntegra em toda a área corpórea. | | | | | | | | | | | | | | | | | | |
| | 2 - Necessidade de cuidados de BAIXA complexidade como: lubrificação da pele, tratamento de dermatites simples, renovação de fixação de cateter venoso periférico. | | | | | | | | | | | | | | | | | | |
| | 3 - Necessidade de cuidados de MÉDIA complexidade como curativos em: feridas limitadas à derme, inserções de drenos, traqueostomia, gastrostomia ou de cateter venoso central. | | | | | | | | | | | | | | | | | | |
| | 4 - Necessidade de cuidados de ALTA complexidade como: desbridamentos, dermatites disseminadas, queimaduras extensas, estomas complexos ou feridas com visualização de fáscia muscular, tecido ósseo ou eviscerações. | | | | | | | | | | | | | | | | | | |

| | Pontuação/Leito | 649A | 649B | 651A | 651B | 653A | 653B | 655A | 655B | 657A | 657B | 659A | 659B | 661A | 661B | 663A | 663B | 663C | 663D |
|---|---|---|---|---|---|---|---|---|---|---|---|---|---|---|---|---|---|---|---|
| Participação do acompanhante | 1 - Não se aplica (não idoso ou não precisa de acompanhante) OU acompanhante reconhece as necessidades físicas e emocionais do paciente e oferece suporte para atendê-las. | | | | | | | | | | | | | | | | | | |
| | 2 - Acompanhante demonstra disponibilidade para incorporar novas informações e habilidades para o cuidado do paciente com participação no planejamento de alta hospitalar. | | | | | | | | | | | | | | | | | | |
| | 3 - Acompanhante demonstra dificuldades ou indisponibilidade para incorporar novas informações e habilidades para o cuidado do paciente E/OU manifesta comportamentos de ansiedade E/OU medo E/OU raiva E/OU retraimento. | | | | | | | | | | | | | | | | | | |
| | 4 - Acompanhante ausente (embora paciente idoso ou com necessidades de acompanhamento permanente) OU acompanhante com impossibilidade física de auxiliar paciente E/OU paciente requer cuidados técnicos de alta complexidade. | | | | | | | | | | | | | | | | | | |
| Rede de apoio e suporte | 1 - Presença de um acompanhante envolvido na prestação e planejamento de cuidados durante todo o tempo OU não se aplica (não idoso ou não precisa de acompanhante). | | | | | | | | | | | | | | | | | | |
| | 2 - Presença de um acompanhante envolvido na prestação e planejamento de cuidados durante mais de 12 horas ao dia. | | | | | | | | | | | | | | | | | | |
| | 3 - Presença de acompanhante envolvido na prestação e planejamento de cuidados durante menos de 12 horas ao dia. | | | | | | | | | | | | | | | | | | |
| | 4 - Ausência de acompanhante OU doença psiquiátrica do acompanhante OU presença de acompanhante que demonstre estresse ou alienação da prestação de cuidados ao paciente. | | | | | | | | | | | | | | | | | | |

**CLASSIFICAÇÃO:** Escore total _____ pontos. **CONSIDERAR:** Cuidados mínimos (11-17 pontos); Cuidados intermediários (18-23 pontos); Alta dependência (24-30 pontos); Cuidados semi intensivos (31-36 pontos); Cuidados intensivos (37-44 pontos).
