# Supplementary material for: Adaptation and validation of an adult patient classification instrument with emphasis on the family dimension
Source: Rev Bras Enferm. 2023 Mar 27;76(2):e20220530. doi: 10.1590/0034-7167-2022-0530 (PMC10042477; doi:10.1590/0034-7167-2022-0530)
Supplement: Supplementary file 4 [file 0034-7167-reben-76-02-e20220530-suppl04.pdf]

**Indicadores**	Juiz 1 (M)	Juiz 2 (E)	Juiz 3 (An)	Juiz 4 (A)	Juiz 5 (G)	Juiz 6 (T)	Juiz 7 (R)
1. Participação do acompanhante	No item 3 interessante colocar que a indisponibilidade para os cuidados é para o ambiente hospitalar. Item 4: Alta hospitalar complexa com vários cuidados que requer vários tratamentos. **I – Relevante/II – Sim.**	Sugiro no item 3 há a possibilidade de incorporar a indisposição do acompanhante em participar da alta hospitalar. **I – Relevante/ II – Sim**	Mesma pontuação dos 2 primeiros itens? **I – Relevante/ II – Sim**	**I – Precisa de pequena revisão/ II – Sim**	**I – Precisa de grande revisão/ II – Não**	I- Pequena revisão<br>II- Não<br>Separa em 2 itens<br>Acrescentar os quais não podem ser realizados pelo acompanhante	Pequena revisão<br>Sim
2. Rede de Apoio e Suporte Familiar	Retirar a palavra pediátrico. **I – Relevante/ II - Sim**	**I – Relevante/ II – Sim**	Retirar pediátrico **I – Relevante/ II – Sim**	**I – Precisa de pequena revisão/ II – Sim**	**I – Precisa de pequena revisão/ II – Sim**	Relevante<br>Sim/Não	Pequena revisão<br>Sim
3. Estado Mental e Atividade	Sugiro colocar intermitente na agitação psicomotora apenas para diferenciar do item 4 pois ao realizar a classificação o profissional achando que é esse estado já marca o item, sendo que após vem a agitação permanente. **I – Relevante/ II – Sim.**	**I – Relevante/ II – Sim.**	No item 4 sugiro acrescentar no final da frase: Insciente ou agitação psicomotora permanente. Necessidade de auxílio e/ou que se faça por ele as atividades diárias. **I – Precisa de pequena revisão/ II –**	**I – Precisa de pequena revisão/ II – Sim**	**I – Relevante/ II – Sim**	Relevante<br>Sim	Pequena revisão<br>Sim

4. Oxigenação	**I – Relevante/ II – Sim**	**I – Relevante/ II – Sim**	**I – Relevante/ II – Sim**	**I – Relevante/ II – Sim**	**I – Relevante/ II – Sim**	Relevante<br>Sim	Grande revisão<br>Não<br>Na definição conceitual, retirar o “normais” e substituir por compatíveis à patologia de base
5. Mobilidade e Deambulação	Órtese e prótese são diferentes, sugiro a colocação dos dois termos.<br>**I – Relevante/ II – Sim.**	**I – Relevante/ II – Sim**	**I – Relevante/ II – Sim**	**I – Relevante/ II – Sim**	**I – Relevante/ II – Sim**	Pequena revisão<br>Sim	Pequena revisão<br>Sim
6. Alimentação e Hidratação	Como este instrumento é proposto para trabalhar em cima de horas de enfermagem, a preparação gasta para realizar alimentação indicadas no item 3 não seria maior que a utilizada para o item 4?<br>**I – Relevante/ II - ?**	Sugiro a inversão do item 4 “nutrição parenteral” para a numeração 3 e a enteral para a numeração 4 devido a demanda de trabalho ser maior no caso da dieta enteral e as complicações possíveis no uso dela (broncoaspiração).<br>**I – Relevante/ II – Não.**	No item 3 substituir a palavra “sonda” por “cateter” nomenclatura mais atual.<br>**I – Precisa de pequena revisão/ II – Sim**	**I – Precisa de pequena revisão/ II – Sim**	**I – Relevante/ II – Sim**	Relevante<br>Sim	Pequena revisão e sim
7. Eliminações	Novamente volto na questão de hora de trabalho, onde penso que após SVD passada ela requer menos tempo em sua manutenção do que você realizar	Sugiro a inversão da pontuação de fralda/comadre para 4 e acredito que esse item deveria ser ser junto do estoma. E a SVD ser pontuado 3. Isso devido a demanda	Item 4 alterar para: cateter vesical/estomas/ Dispositivos para incontinência fecal.<br>**I – Precisa de pequena revisão/ II – Sim**	**I – Relevante/ II – Sim**	**I – Relevante/ II – Sim**	Relevante e sim	Pequena revisão e sim<br>Utiliza vaso...

	várias trocas de fraldas, várias passagens de sonda de alívio, ou colocação de comadre durante os plantões. Ficaria então item 3: SVD e 4: SVA, fralda, comadre, urinol e estomas. **I – Relevante/ II - ?**	de trabalho exigida. **I – Relevante/ II - Não**					
8. Higiene e cuidado corporal	**I – Relevante/ II – Sim**	**I – Relevante/ II – Sim**	O parcial não é claro, deixar banho de aspersão com auxílio. **I – Precisa de pequena revisão/ II – Sim**	**I – Relevante/ II – Sim**	**I – Relevante/ II – Sim**	Relevante e sim	Pequena revisão e sim
9. Intervalo de Aferição de Controles	**I – Relevante/ II – Sim**	**I – Relevante/ II – Sim**	**I – Relevante/ II – Sim**	**I – Relevante/ II – Sim**	**I – Relevante/ II – Sim**	Relevante e sim	Pequena revisão e sim
10. Terapêutica Medicamentosa	Falta da endovenos intermitente e ou contínua as quais a primeira entraria no item 2 e a segunda no item 3. **I – Relevante/ II – Sim**	Sugiro a inversão da pontuação de hemoderivados e/ou quimio de 4 para 3 e o item 3 passa pontuar 4 exatamente pela maior demanda de trabalho **I – Relevante/ II – Não**	Retirar o absoluta e colocar “Indicação e/ou uso de bomba de infusão” **I – Precisa de pequena revisão/ II – Sim**	**I – Precisa de pequena revisão/ II – Sim**	**I – Precisa de pequena revisão/ II – Sim**	Relevante e sim	Relevante e sim
11. Integridade Cutâneo Mucosa	Acrescentar no item 3: fixadores e item 4: feridas complexas ou extensas.	**I – Relevante/ II – Sim**	Acrescentar estomas no item 3 e no item 4 alterar estomas complexos por	**I – Relevante/ II – Sim**	**I – Relevante/ II – Sim**	Relevente e sim Substituir hidratação que é por boca por lubrificação	Relevante e sim

	**I – Relevante/ II - Sim**		estomas com complicações.<br>**I – Precisa de pequena revisão/ II – Sim**				
