# Supplementary material for: Adaptation and validation of an adult patient classification instrument with emphasis on the family dimension
Source: Rev Bras Enferm. 2023 Mar 27;76(2):e20220530. doi: 10.1590/0034-7167-2022-0530 (PMC10042477; doi:10.1590/0034-7167-2022-0530)
Supplement: Supplementary file 5 [file 0034-7167-reben-76-02-e20220530-suppl05.pdf]

Dia	Dia_semana	Leito	Estado Mental e Atividade	Oxigenação	Mobilidade e Deambulação	Alimentação e Hidratação	Eliminações	Higiene e Cuidado Corporal
2	6	633A	1	3	1	1	1	2
2	6	633B	-	-	-	-	-	-
2	6	633C	1	1	2	1	2	3
2	6	633D	1	1	1	1	1	2
2	6	635A	1	1	3	2	3	3
2	6	635B	1	1	1	1	1	2
2	6	637A	1	3	3	2	2	3
2	6	637B	3	1	3	2	2	3
2	6	639A	1	3	4	2	3	4
2	6	639B	-	-	-	-	-	-
2	6	641A	4	4	4	3	3	4
2	6	641B	-	-	-	-	-	-
2	6	643A	3	3	4	2	3	4
2	6	643B	1	1	1	1	1	1
2	6	645A	1	1	2	1	1	2
2	6	645B	1	1	4	2	3	4
2	6	647A	1	1	2	1	1	1
2	6	647B	4	1	4	3	4	4
3	7	633A	1	1	1	1	1	1
3	7	633B	-	-	-	-	-	-
3	7	633C	1	1	1	1	1	2
3	7	633D	1	1	1	1	1	1
3	7	635A	1		3	2	2	3
3	7	635B	1	1	1	1	1	1
3	7	637A	1	3	3	2	2	3
3	7	637B	3	1	3	2	2	4
3	7	639A	1	4	4	2	3	4
3	7	639B	-	-	-	-	-	-
3	7	641A	1	1	3	2	2	3
3	7	641B	-	-	-	-	-	-
3	7	643A	4	3	4	3	3	4
3	7	643B	2	3	4	2	4	4
3	7	645A	1	1		3	1	2
3	7	645B	1	1	3	2	3	3
3	7	647A	1	1	2	1	2	3
3	7	647B	3	1	4	2	3	4
4	1	633A	1	1	3	1	2	3
4	1	633B	-	-	-	-	-	-
4	1	633C	1	1	2	2	2	2
4	1	633D	1	1	1	1	1	3
4	1	635A	1	1	3	2	3	3
4	1	635B	1	1	1	1	1	2
4	1	637A	1	3	3	2	2	3

4	1	637B	3	1	2	2	2	3
4	1	639A	3	4	3	2	3	4
4	1	639B	3	3	3	4	3	4
4	1	641A	-	-	-	-	-	-
4	1	641B	-	-	-	-	-	-
4	1	643A	-	-	-	-	-	-
4	1	643B	3	3	3	3	4	4
4	1	645A	2	1	2	3	2	3
4	1	645B	3	1	3	2	3	3
4	1	647A	1	1	2	1	3	2
4	1	647B	3	1	3	3	4	4
5	2	633A	1	1	3	1	2	2
5	2	633B	1	1	2	1	2	2
5	2	633C	1	1	2	1	2	2
5	2	633D	1	1	1	1	1	2
5	2	635A	1	1	3	2	4	3
5	2	635B	1	1	1	1	1	2
5	2	637A	1	3	3	2	3	3
5	2	637B		1	3	2	3	4
5	2	639A	3	4	3	2	3	4
5	2	639B	1	1	3	2	3	4
5	2	641A	1	1	3	2	4	3
5	2	641B	3	3	3	2	4	4
5	2	643A	1	1	1	1	4	2
5	2	643B	3	3	3	3	4	4
5	2	645A	1	1	2	3	2	2
5	2	645B	-	-	-	-	-	-
5	2	647A	1	1	2	1	2	3
5	3	647B	3	1	4	2	3	4
6	3	633A	1	1	2	1	1	2
6	3	633B	1	1	2	1	1	2
6	3	633C	1	1	2	1	2	2
6	3	633D	1	1	1	1	1	2
6	3	635A	1	1	3	2	4	3
6	3	635B	1	1	1	1	1	2
6	3	637A	1	3	3	2	2	3
6	3	637B	3	1	3	2	2	4
6	3	639A	1	4	3	2	3	4
6	3	639B	-	-	-	-	-	-
6	3	641A	1	1	3	2	2	3
6	3	641B	3	3	3	2	4	4
6	3	643A	1	1	1	1	4	2
6	3	643B	3	3	3	2	4	4
6	3	645A		1	2	1	1	2
6	3	645B	1	1	2	1	1	3
6	3	647A	1	1	2	1	1	3
6	3	647B	3	1	4	2	3	4
7	4	633A	2	1	1	3	1	2
7	4	633B	-	-	-	-	-	-
7	4	633C	2	1	3	2	3	3

7	4	633D	1	1	1	1	1	2
7	4	635A	3	1	3	2	3	3
7	4	635B	3	1	2	1	1	3
7	4	637A	3	3	3	2	3	3
7	4	637B	3	1	3	3	3	3
7	4	639A	3	4	3	3	3	4
7	4	639B	2	1	3	2	3	3
7	4	641A	3	1	3	2	3	3
7	4	641B	3	1	3	2	4	4
7	4	643A	-	-	-	-	-	-
7	4	643B	3	3	3	3	3	3
7	4	645A	3	1	2	2	2	3
7	4	645B	3	1	3	1	3	3
7	4	647A	2	1	2	2	3	3
7	4	647B	2	2	2	3	2	3
8	5	633A	2	2	1	4	2	3
8	5	633B	3	3	3	2	3	3
8	5	633C	2	2	2	2	2	3
8	5	633D	2	1	2	1	2	3
8	5	635A	2	2	3	2	2	3
8	5	635B	2	2	2	2	2	3
8	5	637A	-	-	-	-	-	-
8	5	637B	2	2	2	1	3	3
8	5	639A	-	-	-	-	-	-
8	5	639B	3	3	3	3	3	4
8	5	641A	-	-	-	-	-	-
8	5	641B	3	2	4	2	3	4
8	5	643A	3	3	3	2	3	3
8	5	643B	3	2	3	3	3	3
8	5	645A	3	2	2	2	2	2
8	5	645B	3	2	3	1	3	3
8	5	647A	2	2	2	1	3	3
8	5	647B	2	2	2	1	2	2
9	6	633A	-	-	-	-	-	-
9	6	633B	-	-	-	-	-	-
9	6	633C	2	2	2	2	2	3
9	6	633D	-	-	-	-	-	-
9	6	635A	2	2	3	2	2	3
9	6	635B	2	2	2	4	2	3
9	6	637A	2	2	3	1	2	3
9	6	637B	2	2	2	2	2	3
9	6	639A	2	1	1	2	1	2
9	6	639B	2	3	3	4	3	3
9	6	641A	3	3	4	4	4	4
9	6	641B	3	2	2	4	2	3
9	6	643A	3	3	3	2	3	3
9	6	643B	3	2	3	4	3	3
9	6	645A	3	2	2	2	2	2
9	6	645B	3	2	2	4	3	3
9	6	647A	2	2	3	4	3	3

9	6	647B	2	2	2	4	2	2
10	7	633A	2	2	1	1	1	2
10	7	633B	2	2	2	1	2	2
10	7	633C	2	2	1	1	1	2
10	7	633D	-	-	-	-	-	-
10	7	635A	2	2	2	1	2	3
10	7	635B	2	2	1	1	1	2
10	7	637A	3	2	3	2	3	3
10	7	637B	3	3	1	3	1	3
10	7	639A	3	3	3	3	3	3
10	7	639B	2	2	1	3	1	2
10	7	641A	3	4	3	3	4	4
10	7	641B	2	2	2	2	3	3
10	7	643A	3	3	3	2	3	3
10	7	643B	3	2	3	2	3	3
10	7	645A	3	4	3	3	3	4
10	7	645B	-	-	-	-	-	-
10	7	647A	3	2	3	2	3	3
10	7	647B	3	2	2	2	2	2
11	1	633A	-	-	-	-	-	-
11	1	633B	2	2	2	1	2	3
11	1	633C	2	2	2	1	1	2
11	1	633D	-	-	-	-	-	-
11	1	635A	3	2	3	1	3	3
11	1	635B	2	2	1	1	1	2
11	1	637A	-	-	-	-	-	-
11	1	637B	3	2	1	3	1	3
11	1	639A	3	3	3	3	3	3
11	1	639B	2	2	1	1	1	2
11	1	641A	3	4	4	3	4	4
11	1	641B	3	2	3	2	3	3
11	1	643A	3	3	3	3	3	3
11	1	643B	3	2	3	3	3	3
11	1	645A	3	2	3	3	3	4
11	1	645B	3	2	3	1	3	3
11	1	647A	3	4	3	2	3	3
11	1	647B	2	2	2	2	2	2
12	2	633A	-	-	-	-	-	-
12	2	633B	2	1	2	1	2	3
12	2	633C	1	1	1	1	2	2
12	2	633D	-	-	-	-	-	-
12	2	635A	1	1	2	1	2	3
12	2	635B	1	1	2	1	2	3
12	2	637A	-	-	-	-	-	-
12	2	637B	1	2	2	3	2	3
12	2	639A	2	3	3	3	3	4
12	2	639B	1	1	3	4	2	3
12	2	641A	2	3	4	2	4	4
12	2	641B	1	2	3	1	3	3
12	2	643A	1	3	3	2	2	3

12	2	643B	1	2	3	2	3	3
12	2	645A	3	2	3	2	3	3
12	2	645B	1	1	2	1	2	3
12	2	647A	1	1	2	1	2	3
12	3	647B	1	2	2	1	2	3
13	3	633A	3	2	3	2	3	3
13	3	633B	-	-	-	-	-	-
13	3	633C	2	2	1	1	1	2
13	3	633D	2	2	1	1	1	2
13	3	635A	3	2	3	2	3	3
13	3	635B	2	2	1	1	1	2
13	3	637A	3	2	3	2	3	3
13	3	637B	3	3	1	3	1	2
13	3	639A	3	3	4	3	3	4
13	3	639B	3	2	1	1	1	2
13	3	641A	3	4	4	3	4	4
13	3	641B	3	2	3	3	3	3
13	3	643A	3	3	3	3	3	3
13	3	643B	3	2	3	2	3	3
13	3	645A	3	3	3	3	3	3
13	3	645B	3	2	3	1	3	3
13	3	647A	3	2	3	1	3	3
13	3	647B	3	2	3	2	3	3
14	4	633A	3	2	3	2	3	3
14	4	633B	3	3	3	3	3	3
14	4	633C	2	2	1	1	1	1
14	4	633D	2	2	1	1	1	1
14	4	635A	3	2	3	2	3	3
14	4	635B	2	2	1	1	1	2
14	4	637A	1	2	1	1	1	1
14	4	637B	1	3	1	1	1	2
14	4	639A	1	3	4	2	4	4
14	4	639B	-	-	-	-	-	-
14	4	641A	1	2	4	2	4	4
14	4	641B	3	3	4	4	4	4
14	4	643A	1	3	3	2	3	3
14	4	643B	3	2	3	2	3	3
14	4	645A	3	2	3	2	3	3
14	4	645B	1	2	3	2	3	3
14	4	647A	1	3	3	1	3	3
14	4	647B	1	2	3	1	3	3
15	5	633A	3	2	3	2	3	3
15	5	633B	3	3	3	3	3	3
15	5	633C	-	-	-	-	-	-
15	5	633D	2	1	1	1	1	2
15	5	635A	3	1	3	2	3	3
15	5	635B	3	1	3	2	3	3
15	5	637A	-	-	-	-	-	-
15	5	637B	3	1	4	2	4	4
15	5	639A	3	3	4	3	3	4

15	5	639B	3	2	3	2	3	3
15	5	641A	3	1	4	2	3	4
15	5	641B	4	3	4	4	4	4
15	5	643A	3	3	3	3	3	3
15	5	643B	-	-	-	-	-	-
15	5	645A	3	2	3	2	3	3
15	5	645B	3	1	4	2	3	3
15	5	647A	-	-	-	-	-	-
15	5	647B	3	3	3	2	3	3
16	6	633A	3	1	3	2	3	3
16	6	633B	1	3	3	2	3	3
16	6	633C	3	1	3	2	3	3
16	6	633D	-	-	-	-	-	-
16	6	635A	1	1	3	1	3	3
16	6	635B	-	-	-	-	-	-
16	6	637A	2	1	4	2	4	4
16	6	637B	2	1	4	2	4	4
16	6	639A	3	1	4	3	3	4
16	6	639B	1	4	3	2	3	3
16	6	641A	3	1	4	3	4	4
16	6	641B	3	3	4	3	3	4
16	6	643A	1	3	3	2	3	3
16	6	643B	1	1	3	2	2	3
16	6	645A	3	1	4	3	3	4
16	6	645B	1	1	3	2	2	3
16	6	647A	1	1	2	2	2	3
16	6	647B	1	1	2	2	2	3
17	7	633A	1	1	2	1	3	3
17	7	633B	2	3	3	2	3	3
17	7	633C	3	1	3	2	3	4
17	7	633D	1	1	2	1	3	3
17	7	635A	1	1	3	1	3	3
17	7	635B	-	-	-	-	-	-
17	7	637A	-	-	-	-	-	-
17	7	637B	2	3	3	2	4	4
17	7	639A	2	1	4	3	3	4
17	7	639B	1	4	2	1	3	3
17	7	641A	3	1	4	2	4	4
17	7	641B	3	3	4	4	4	4
17	7	643A	1	3	2	1	3	3
17	7	643B	1	1	1	1	3	3
17	7	645A	3	1	4	3	4	4
17	7	645B	2	1	3	2	4	4
17	7	647A	1	1	2	1	2	3
17	7	647B	1	1	2	1	2	3
18	1	633A	1	1	2	1	3	3
18	1	633B	1	3	3	2	3	3
18	1	633C	2	1	3	2	3	3
18	1	633D	-	-	-	-	-	-
18	1	635A	3	1	3	2	3	4

18	1	635B	1	1	2	1	3	3
18	1	637A	-	-	-	-	-	-
18	1	637B	2	3	3	2	3	4
18	1	639A	2	1	4	2	3	4
18	1	639B	1	4	3	1	3	3
18	1	641A	-	-	-	-	-	-
18	1	641B	3	3	4	4	4	4
18	1	643A	2	3	3	1	3	3
18	1	643B	1	1	2	1	2	3
18	1	645A	3	1	4	3	4	4
18	1	645B	2	1	3	2	4	4
18	1	647A	1	1	2	1	2	2
18	1	647B	1	1	2	1	2	2
19	2	633A	3		2	1	3	2
19	2	633B	3	3	3	3	3	3
19	2	633C	-	-	-	-	-	-
19	2	633D	2	1	1	1	1	2
19	2	635A	3	2	4	3	3	4
19	2	635B	2	2	2	1	1	2
19	2	637A	-	-	-	-	-	-
19	2	637B	3	2	3	2	4	3
19	2	639A	3	3	4	3	3	4
19	2	639B	3	2	3	2	3	3
19	2	641A	-	-	-	-	-	-
19	2	641B	4	3	4	4	4	4
19	2	643A	3	2	3	3	3	3
19	2	643B	3	2	3	2	3	3
19	2	645A	4	2	4	3	4	4
19	2	645B	3	2	4	4	4	4
19	2	647A	3	2	3	2	2	3
19	3	647B	3	2	3	3	3	3
20	3	633A	1	1	2	1	3	2
20	3	633B	1	3	3	2	3	4
20	3	633C	-	-	-	-	-	-
20	3	633D	-	-	-	-	-	-
20	3	635A	3	1	3	2	3	4
20	3	635B	1	1	1	1	1	2
20	3	637A	-	-	-	-	-	-
20	3	637B	1	2	3	1	4	3
20	3	639A	3	1	4	3	3	4
20	3	639B	2	4	3	1	3	3
20	3	641A	-	-	-	-	-	-
20	3	641B	3	3	4	4	4	4
20	3	643A	2	3	3	1	3	3
20	3	643B	1	1	2	1	2	2
20	3	645A	3	2	4	3	4	4
20	3	645B	-	-	-	-	-	-
20	3	647A	1	1	2	1	2	2
20	3	647B	1	3	2	1	2	2
21	4	633A	2	2	2	2	3	3

21	4	633B	3	3	3	3	3	4
21	4	633C	-	-	-	-	-	-
21	4	633D	3	2	3	4	3	3
21	4	635A	-	-	-	-	-	-
21	4	635B	2	1	2	1	2	2
21	4	637A	3	1	3	2	3	3
21	4	637B	3	1	3	2	4	3
21	4	639A	3	3	4	3	3	4
21	4	639B	3	4	4	3	3	4
21	4	641A	3	1	4	3		
21	4	641B	-	-	-	-	-	-
21	4	643A	3	3	3	3	3	3
21	4	643B	2	1	2	2	2	3
21	4	645A	3	3	4	3	4	4
21	4	645B	-	-	-	-	-	-
21	4	647A	2	1	2	1	2	3
21	4	647B	2	2	2	1	2	3
22	5	633A	1	1	2	1	3	2
22	5	633B	1	3	3	1	3	3
22	5	633C	2	1	3	1	3	4
22	5	633D	-	-	-	-	-	-
22	5	635A	-	-	-	-	-	-
22	5	635B	1	1	2	1	2	2
22	5	637A	3	2	4	3	4	4
22	5	637B	2	3	3	1	4	3
22	5	639A	3	4	3	2	3	3
22	5	639B	-	-	-	-	-	-
22	5	641A	-	-	-	-	-	-
22	5	641B	-	-	-	-	-	-
22	5	643A	2	3	3	1	3	3
22	5	643B	1	1	2	1	2	2
22	5	645A	3	1	4	2	4	4
22	5	645B	1	1	3	1	2	3
22	5	647A	1	1	2	1	2	2
22	5	647B	1	1	2	1	2	2
23	6	633A	3	1	2	1	3	2
23	6	633B	3	3	3	3	3	3
23	6	633C	-	-	-	-	-	-
23	6	633D	3	1	3	2	3	3
23	6	635A	3	1	3	2	3	3
23	6	635B	2	1	2	1	1	2
23	6	637A	4	3	4	4	4	4
23	6	637B	3	1	3	2	4	3
23	6	639A	3	2	3	3	3	3
23	6	639B	3	1	3	2	3	3
23	6	641A	-	-	-	-	-	-
23	6	641B	-	-	-	-	-	-
23	6	643A	3	3	3	3	3	3
23	6	643B	3	1	2	1	1	3
23	6	645A	3	1	4	3	4	4

23	6	645B	2	1	2	1	1	3
23	6	647A	3	1	2	1	2	2
23	6	647B	3	3	2	2	2	2
24	7	633A	3	1	2	1	3	2
24	7	633B	3	3	3	3	3	3
24	7	633C	-	-	-	-	-	-
24	7	633D	3	1	3	1	3	3
24	7	635A	3	1	3	2	3	3
24	7	635B	2	1	2	1	1	2
24	7	637A	4	3	4	4	4	4
24	7	637B	3	1	3	2	4	3
24	7	639A	3	2	3	2	3	3
24	7	639B	3	1	3	2	3	3
24	7	641A	-	-	-	-	-	-
24	7	641B	3	1	3	2	3	3
24	7	643A	-	-	-	-	-	-
24	7	643B	3	1	2	2	2	3
24	7	645A	3	1	4	2	4	4
24	7	645B	-	-	-	-	-	-
24	7	647A	-	-	-	-	-	-
24	7	647B	2	1	2	1	2	3
25	1	633A	3	1	2	1	3	3
25	1	633B	3	3	3	3	3	3
25	1	633C	-	-	-	-	-	-
25	1	633D	3	1	3	2	3	3
25	1	635A	3	1	3	2	3	3
25	1	635B	2	1	2	1	1	2
25	1	637A	4	1	4	4	3	4
25	1	637B	3	1	3	2	4	3
25	1	639A	3	4	3	2	3	3
25	1	639B	3	1	3	2	3	3
25	1	641A	3	3	4	4	3	4
25	1	641B	-	-	-	-	-	-
25	1	643A	3	1	3	1	3	3
25	1	643B	3	1	2	1	1	2
25	1	645A	-	-	-	-	-	-
25	1	645B	3	1	3	1	3	3
25	1	647A	-	-	-	-	-	-
25	1	647B	3	1	3	1	3	3
26	2	633A	1	1	2	1	3	2
26	2	633B	1	3	3	1	3	3
26	2	633C	-	-	-	-	-	-
26	2	633D	2	1	3	1	3	3
26	2	635A	2	1	3	1	3	3
26	2	635B	1	1	1	1	2	2
26	2	637A	3	3	4	3	4	4
26	2	637B	-	-	-	-	-	-
26	2	639A	2	4	3	1	3	3
26	2	639B	2	1	3	1	3	3
26	2	641A	3	1	3	3	3	4

26	2	641B	-	-	-	-	-	-
26	2	643A	1	1	2	1	2	3
26	2	643B	1	1	2	1	2	3
26	2	645A	2	3	2	1	3	3
26	2	645B	1	1	2	1	2	3
26	2	647A	-	-	-	-	-	-
26	3	647B	1	1	1	1	1	1
27	3	633A	1	1	1	1	1	2
27	3	633B	1	1	3	1	3	3
27	3	633C	1	1	1	1	1	2
27	3	633D	1	1	3	1	3	3
27	3	635A	1	1	3	2	3	3
27	3	635B	1	1	1	1	1	1
27	3	637A	3	3	4	3	4	4
27	3	637B	1	1	2	1	2	3
27	3	639A	1	4	3		3	3
27	3	639B	1	1	3		3	3
27	3	641A	1	1	2	2	2	2
27	3	641B	-	-	-	-	-	-
27	3	643A	1	1	2	1	2	2
27	3	643B	1	1	2	1	2	2
27	3	645A	1	3	2	1	2	3
27	3	645B	1	1	2	1	2	3
27	3	647A	1	1	1	1	1	1
27	3	647B	1	1	1	1	1	1

Intervalo de Aferição de Controles	Terapêutica Medicamentosa	Integridade Cutâneo Mucosa	Participação do acompanhante	Rede de apoio e suporte	TOTAL	Categoria de Cuidado
2	2	1	1	2	17	Cuidados Mínimos
-	-	-	-	-	0	Vago
1	3	2	2	2	20	Cuidados Intermediários
1	3	3	1	1	16	Cuidados Mínimos
1	2	3	2	3	24	Alta Dependência
1	3	3	1	1	16	Cuidados Mínimos
1	3	2	4	3	27	Alta Dependência
1	3	3	2	2	25	Alta Dependência
1	3	3	4	4	32	Cuidados Semi Intensivos
-	-	-	-	-	0	Vago
4	4	4	4	4	42	Cuidados Intensivos
-	-	-	-	-	0	Vago
1	3	2	4	4	33	Cuidados Semi Intensivos
1	2	1	2	3	15	Cuidados Mínimos
1	2	2	4	4	21	Cuidados Intermediários
1	3	3	2	2	26	Alta Dependência
1	3	2	1	1	15	Cuidados Mínimos
1	4	3	3	2	33	Cuidados Semi Intensivos
1	2	1	1	2	13	Cuidados Mínimos
-	-	-	-	-	0	Vago
1	3	2	2	2	17	Cuidados Mínimos
1	3	2	1	1	14	Cuidados Mínimos
2	4	3	2	3	25	Alta Dependência
1	3	2	1	1	14	Cuidados Mínimos
1	3	2	4	3	27	Alta Dependência
1	3	2	2	1	24	Alta Dependência
4	3	3	4	4	36	Cuidados Semi Intensivos
-	-		-	-	0	Vago
4	3	3	2	3	27	Alta Dependência
-	-	-	-	-	0	Vago
1	4	2	4	4	36	Cuidados Semi Intensivos
4	3	2	2	3	33	Cuidados Semi Intensivos
1	3	1	4	4	21	Cuidados Intermediários
1	3	2	4	2	25	Alta Dependência
1	3	2	1	1	18	Cuidados Intermediários
1	4	2	3	2	29	Alta Dependência
1	2	2	1	1	18	Cuidados Intermediários
-	-	-	-	-	0	Vago
1	3	2	2	2	20	Cuidados Intermediários
1	3	3	1	1	17	Cuidados Mínimos
1	3	2	4	4	27	Alta Dependência
1	3	2	1	1	15	Cuidados Mínimos
1	3	2	1	1	22	Cuidados Intermediários

1	3	2	3	2	24	Alta Dependência
4	3	3	4	4	37	Cuidados Intensivos
2	3	3	2	2	32	Cuidados Semi Intensivos
-	-	-	-	-	0	Vago
-	-	-	-	-	0	Vago
-	-	-	-	-	0	Vago
4	4	3	2	2	35	Cuidados Semi Intensivos
1	3	2	1	1	21	Cuidados Intermediários
1	3	3	4	4	30	Alta Dependência
1	3	2	1	1	18	Cuidados Intermediários
4	4	3	2	2	33	Cuidados Semi Intensivos
1	2	1	2	1	17	Cuidados Mínimos
1	3	2	2	2	19	Cuidados Intermediários
1	3	2	2	2	19	Cuidados Intermediários
1	3	3	1	1	16	Cuidados Mínimos
1	3	3	4	4	29	Alta Dependência
1	3	3	1	1	16	Cuidados Mínimos
1	3	2	1	1	23	Cuidados Intermediários
1	3	2	3	2	24	Alta Dependência
4	3	2	4	4	36	Cuidados Semi Intensivos
2	3	2	2	2	25	Alta Dependência
1	3	3	4	4	29	Alta Dependência
4	3	3	2	2	33	Cuidados Semi Intensivos
1	3	2	1	1	18	Cuidados Intermediários
4	4	3	2	2	35	Cuidados Semi Intensivos
1	2	2	4	4	24	Alta Dependência
-	-	-	-	-	0	Vago
1	3	2	1	1	18	Cuidados Intermediários
4	4	3	3	4	35	Cuidados Semi Intensivos
1	2	1	2	1	15	Cuidados Mínimos
1	3	2	2	2	18	Cuidados Intermediários
1	3	2	2	2	19	Cuidados Intermediários
1	3	3	1	1	16	Cuidados Mínimos
1	3	3	4	4	29	Alta Dependência
1	3	3	1	1	16	Cuidados Mínimos
1	3	2	1	1	22	Cuidados Intermediários
1	3	2	3	2	26	Alta Dependência
4	3	2	4	4	34	Cuidados Semi Intensivos
-	-	-	-	-	0	Vago
1	3	3	4	4	27	Alta Dependência
4	3	3	2	2	33	Cuidados Semi Intensivos
1	3	3	1	1	19	Cuidados Intermediários
4	4	3	2	2	34	Cuidados Semi Intensivos
1	3	2	4	4	21	Cuidados Intermediários
1	3	3	1	1	18	Cuidados Intermediários
1	3	2	1	1	17	Cuidados Mínimos
4	4	3	3	4	35	Cuidados Semi Intensivos
1	3	2	1	1	18	Cuidados Intermediários
-	-	-	-	-	0	Vago
1	3	2	2	2	24	Alta Dependência

1	3	3	1	1	16	Cuidados Mínimos
1	3	2	4	4	29	Alta Dependência
1	3	3	1	1	20	Cuidados Intermediários
1	3	2	1	1	25	Alta Dependência
1	3	2	3	3	28	Alta Dependência
4	3	2	4	4	37	Cuidados Intensivos
1	3	3	2	2	25	Alta Dependência
1	3	2	4	4	29	Alta Dependência
2	3	2	2	2	28	Alta Dependência
-	-	-	-	-	0	Vago
1	3	2	4	4	32	Cuidados Semi Intensivos
1	3	2	1	1	21	Cuidados Intermediários
1	3	3	1	1	23	Cuidados Intermediários
2	4	2	1	1	23	Cuidados Intermediários
1	3	2	1	1	22	Cuidados Intermediários
1	3	2	1	1	22	Cuidados Intermediários
1	3	2	4	4	31	Cuidados Semi Intensivos
2	3	3	2	2	25	Alta Dependência
1	3	3	1	1	20	Cuidados Intermediários
1	3	2	4	4	28	Alta Dependência
1	3	3	1	1	22	Cuidados Intermediários
-	-	-	-	-	0	Vago
1	3	2	4	4	27	Alta Dependência
-	-	-	-	-	0	Vago
2	3	2	4	4	34	Cuidados Semi Intensivos
-	-	-	-	-	0	Vago
2	3	3	4	4	34	Cuidados Semi Intensivos
2	3	2	1	1	26	Alta Dependência
1	3	2	2	2	27	Alta Dependência
1	3	2	1	1	21	Cuidados Intermediários
1	3	3	1	1	24	Alta Dependência
4	4	2	1	1	25	Alta Dependência
1	3	2	1	1	19	Cuidados Intermediários
-	-	-	-	-	0	Vago
-	-	-	-	-	0	Vago
1	3	3	2	2	24	Alta Dependência
-	-	-	-	-	0	Vago
1	3	2	4	4	28	Alta Dependência
1	3	3	1	1	24	Alta Dependência
1	3	2	1	2	22	Cuidados Intermediários
1	3	3	4	1	25	Alta Dependência
1	3	2	4	4	23	Cuidados Intermediários
2	3	2	4	4	33	Cuidados Semi Intensivos
2	3	2	2	2	33	Cuidados Semi Intensivos
1	3	2	4	4	30	Alta Dependência
2	3	2	4	1	29	Alta Dependência
1	3	2	2	2	28	Alta Dependência
1	3	2	1	1	21	Cuidados Intermediários
1	3	3	1	1	26	Alta Dependência
4	4	2	1	1	29	Alta Dependência

1	3	2	1	1	22	Cuidados Intermediários
1	3	2	1	1	17	Cuidados Mínimos
1	3	2	2	2	21	Cuidados Intermediários
2	3	2	1	1	18	Cuidados Intermediários
-	-	-	-	-	0	Vago
1		2	4	4	23	Cuidados Intermediários
1	3	3	1	1	18	Cuidados Intermediários
1	3	3	2	2	27	Alta Dependência
1	3	3	4	4	29	Alta Dependência
4	3	2	4	4	35	Cuidados Semi Intensivos
1	3	2	4	4	25	Alta Dependência
2	3	2	2	3	33	Cuidados Semi Intensivos
2	3	2	1	1	23	Cuidados Intermediários
2	3	2	1	1	26	Alta Dependência
1	4	2	2	2	27	Alta Dependência
2	3	2	4	4	35	Cuidados Semi Intensivos
-	-	-	-	-	0	Vago
4	4	2	1	1	28	Alta Dependência
2	3	2	1	1	22	Cuidados Intermediários
-	-	-	-	-	0	Vago
1	3	2	2	2	22	Cuidados Intermediários
1	3	2	1	1	18	Cuidados Intermediários
-	-	-	-	-	0	Vago
2	2	2	4	4	29	Alta Dependência
1	3	3	1	1	18	Cuidados Intermediários
-	-	-	-	-	0	Vago
1	3	3	4	4	28	Alta Dependência
4	3	2	4	4	35	Cuidados Semi Intensivos
1	3	2	4	4	23	Cuidados Intermediários
4	3	2	2	3	36	Cuidados Semi Intensivos
2	3	2	1	1	25	Alta Dependência
2	2	2	1	1	26	Alta Dependência
1	3	2	2	1	26	Alta Dependência
2	3	2	4	4	33	Cuidados Semi Intensivos
1	3	3	4	4	30	Alta Dependência
4	4	2	1	1	30	Alta Dependência
1	2	2	1	1	19	Cuidados Intermediários
-	-	-	-	-	0	Vago
1	3	3	4	4	26	Alta Dependência
1	3	2	4	4	22	Cuidados Intermediários
-	-	-	-	-	0	Vago
1	3	2	4	4	24	Alta Dependência
1	3	3	4	4	25	Alta Dependência
-	-	-	-	-	0	Vago
1	3	3	4	4	28	Alta Dependência
4	3	1	4	4	34	Cuidados Semi Intensivos
1	4	3	4	4	30	Alta Dependência
4	3	2	4	4	36	Cuidados Semi Intensivos
1	3	2	4	4	27	Alta Dependência
1	3	2	4	4	28	Alta Dependência

1	3	2	2	2	24	Alta Dependência
1	3	2	4	4	30	Alta Dependência
1	3	3	4	4	25	Alta Dependência
4	4		4	4	26	Alta Dependência
1	3		4	4	23	Cuidados Intermediários
2	3	2	1	1	25	Alta Dependência
-	-	-	-	-	0	Vago
1	3	2	1	1	17	Cuidados Mínimos
1	3	2	4	4	23	Cuidados Intermediários
1	3	2	4	4	30	Alta Dependência
1	3	3	1	1	18	Cuidados Intermediários
1	3	2	1	1	24	Alta Dependência
1	3	3	4	4	28	Alta Dependência
4	3	2	4	4	37	Cuidados Intensivos
1	3	3	1	1	19	Cuidados Intermediários
4	3	2	1	1	33	Cuidados Semi Intensivos
4	3	2	1	1	28	Alta Dependência
2	3	2	2	2	29	Alta Dependência
1	3	2	2	2	26	Alta Dependência
2	3	2	4	4	33	Cuidados Semi Intensivos
1	3	3	2	2	26	Alta Dependência
4	4	2	1	1	27	Alta Dependência
1	3	2	1	1	24	Alta Dependência
2	3	2	1	1	25	Alta Dependência
4	3	2	4	4	35	Cuidados Semi Intensivos
1	3	2	1	1	16	Cuidados Mínimos
1	3	2	4	4	22	Cuidados Intermediários
1	3	2	4	4	30	Alta Dependência
1	3	3	1	1	18	Cuidados Intermediários
1	3	2	1	1	15	Cuidados Mínimos
1	3	3	4	4	24	Alta Dependência
4	3	2	4	4	35	Cuidados Semi Intensivos
-	-	-	-	-	0	Vago
4	3	2	4	4	34	Cuidados Semi Intensivos
4	4	2	2	2	36	Cuidados Semi Intensivos
4	3	2	2	2	28	Alta Dependência
1	3	2	2	2	26	Alta Dependência
1	3	2	4	4	30	Alta Dependência
1	3	3	2	2	25	Alta Dependência
4	4	2	4	4	32	Cuidados Semi Intensivos
1	3	2	4	1	24	Alta Dependência
1	3	2	1	1	24	Alta Dependência
4	3	2	4	4	35	Cuidados Semi Intensivos
-	-	-	-	-	0	Vago
1	2	2	4	4	21	Cuidados Intermediários
2	3	2	4	4	30	Alta Dependência
1	3	3	1	1	24	Alta Dependência
-	-	-	-	-	0	Vago
1	3	1	1	1	25	Alta Dependência
4	3	3	4	4	38	Cuidados Intensivos

4	4	2	1	1	28	Alta Dependência
1	3	3	3	2	29	Alta Dependência
4	4	3	2	2	38	Cuidados Intensivos
1	3	2	1	1	26	Alta Dependência
-	-	-	-	-	0	Vago
1	3	2	4	4	30	Alta Dependência
1	3	3	2	2	27	Alta Dependência
-	-	-	-	-	0	Vago
2	3	2	1	1	26	Alta Dependência
1	3	2	4	4	29	Alta Dependência
4	3	2	4	4	32	Cuidados Semi Intensivos
1	3	3	4	4	30	Alta Dependência
-	-	-	-	-	0	Vago
1	3	3	4	4	27	Alta Dependência
-	-	-	-	-	0	Vago
1	3	3	4	4	32	Cuidados Semi Intensivos
1	3	3	4	4	32	Cuidados Semi Intensivos
1	3	3	2	3	30	Alta Dependência
4	4	2	4	4	34	Cuidados Semi Intensivos
4	3	3	2	3	34	Cuidados Semi Intensivos
4	4	3	2	2	35	Cuidados Semi Intensivos
1	3	2	4	4	29	Alta Dependência
1	3	2	4	4	26	Alta Dependência
1	3	3	2	2	29	Alta Dependência
1	3	3	2	3	24	Alta Dependência
	3	2	4	4	24	Alta Dependência
	3	2	4	4	24	Alta Dependência
1	3	2	4	4	25	Alta Dependência
4	3	2	4	4	33	Cuidados Semi Intensivos
1	3	3	4	4	31	Cuidados Semi Intensivos
1	3	2	4	4	25	Alta Dependência
1	3	3	4	4	27	Alta Dependência
-	-	-	-	-	0	Vago
-	-	-	-	-	0	Vago
1	3	3	4	4	33	Cuidados Semi Intensivos
1	3	3	4	4	32	Cuidados Semi Intensivos
4	4	3	4	4	33	Cuidados Semi Intensivos
4	3	3	2	3	33	Cuidados Semi Intensivos
4	4	3	2	3	38	Cuidados Intensivos
1	3	2	4	4	27	Alta Dependência
1	3	2	4	4	24	Alta Dependência
1	3	3	2	3	31	Cuidados Semi Intensivos
1	3	3	2	3	28	Alta Dependência
	3	3	4	4	24	Alta Dependência
	3	2	4	4	23	Cuidados Intermediários
	3	2	4	4	24	Alta Dependência
	3	2	4	4	28	Alta Dependência
	3	3	2	3	25	Alta Dependência
-	-	-	-	-	0	Vago
1	3	3	4	4	31	Cuidados Semi Intensivos

1	3	2	4	4	25	Alta Dependência
-	-	-	-	-	0	Vago
1	3	3	4	4	32	Cuidados Semi Intensivos
1	3	3	4	4	31	Cuidados Semi Intensivos
4	3	2	4	4	32	Cuidados Semi Intensivos
-	-	-	-	-	0	Vago
4	4	3	2	2	37	Cuidados Intensivos
1	3	2	4	4	29	Alta Dependência
1	3	2	4	4	24	Alta Dependência
	3	3	2	3	30	Alta Dependência
	3	3	2	3	27	Alta Dependência
1	3	3	4	4	24	Alta Dependência
1	3	2	4	4	23	Cuidados Intermediários
1	3	2	1	1	19	Cuidados Intermediários
2	3	2	4	4	33	Cuidados Semi Intensivos
-	-	-	-	-	0	Vago
1	1	2	2	2	16	Cuidados Mínimos
2	3	4	3	2	33	Cuidados Semi Intensivos
2	3	2	1	1	19	Cuidados Intermediários
-	-	-	-	-	0	Vago
1	3	2	1	1	25	Alta Dependência
4	3	4	4	4	39	Cuidados Intensivos
4	4	2	4	4	34	Cuidados Semi Intensivos
-	-	-	-	-	0	Vago
4	4	3	2	2	38	Cuidados Intensivos
2	3	2	1	1	26	Alta Dependência
1	3	2	4	4	30	Alta Dependência
2	3	2	2	2	32	Cuidados Semi Intensivos
2	3	2	2	2	32	Cuidados Semi Intensivos
1	3	2	1	1	23	Cuidados Intermediários
1	3	2	1	1	25	Alta Dependência
1	3	2	4	4	24	Alta Dependência
1	3	2	4	4	30	Alta Dependência
-	-	-	-	-	0	Vago
-	-	-	-	-	0	Vago
1	3	3	2	3	28	Alta Dependência
1	3	2	4	4	21	Cuidados Intermediários
-	-	-	-	-	0	Vago
1	3	3	4	4	29	Alta Dependência
1	3	3	4	4	33	Cuidados Semi Intensivos
4	4	2	4	4	34	Cuidados Semi Intensivos
-	-	-	-	-	0	Vago
4	4	3	2	2	37	Cuidados Intensivos
1	3	2	4	4	29	Alta Dependência
1	3	2	4	4	23	Cuidados Intermediários
1	3	2	2	3	31	Cuidados Semi Intensivos
-	-	-	-	-	0	Vago
1	3	2	4	4	23	Cuidados Intermediários
1	3	2	4	4	25	Alta Dependência
1	3	2	1	1	22	Cuidados Intermediários

2	3	2	4	4	34	Cuidados Semi Intensivos
-	-	-	-	-	0	Vago
1	3	3	4	4	33	Cuidados Semi Intensivos
-	-	-	-	-	0	Vago
2	3	2	1	1	19	Cuidados Intermediários
1	3	3	4	4	30	Alta Dependência
1	3	3	1	1	25	Alta Dependência
2	4	4	4	4	38	Cuidados Intensivos
4	4	2	4	4	39	Cuidados Intensivos
			1	1	13	Cuidados Mínimos
-	-	-	-	-	0	Vago
2	3	2	1	1	27	Alta Dependência
1	3	2	4	4	26	Alta Dependência
1	3	2	2	2	31	Cuidados Semi Intensivos
-	-	-	-	-	0	Vago
1	3	3	1	1	20	Cuidados Intermediários
1	3	2	1	1	20	Cuidados Intermediários
1	3	2	4	4	24	Alta Dependência
1	3	2	4	4	28	Alta Dependência
1	3	3	4	4	29	Alta Dependência
-	-	-	-	-	0	Vago
-	-	-	-	-	0	Vago
1	3	2	4	4	23	Cuidados Intermediários
1	3	2	3	3	32	Cuidados Semi Intensivos
1	3	2	4	4	30	Alta Dependência
4	4	2	4	4	36	Cuidados Semi Intensivos
-	-	-	-	-	0	Vago
-	-	-	-	-	0	Vago
-	-	-	-	-	0	Vago
1	3	2	4	4	29	Alta Dependência
1	3	2	4	4	23	Cuidados Intermediários
1	3	3	2	3	30	Alta Dependência
1	3	3	4	4	26	Alta Dependência
1	3	3	4	4	24	Alta Dependência
1	3	2	4	4	23	Cuidados Intermediários
1	3	2	1	1	20	Cuidados Intermediários
2	3	2	4	4	33	Cuidados Semi Intensivos
-	-	-	-	-	0	Vago
1	3	3	4	4	30	Alta Dependência
1	3	3	4	4	30	Alta Dependência
1	3	2	1	1	17	Cuidados Mínimos
2	3	2	2	2	34	Cuidados Semi Intensivos
1	3	2	1	1	24	Alta Dependência
4	4	2	1	1	29	Alta Dependência
1	3	2	2	2	25	Alta Dependência
-	-	-	-	-	0	Vago
-	-	-	-	-	0	Vago
2	3	2	1	1	27	Alta Dependência
1	3	2	1	1	19	Cuidados Intermediários
4	3	3	1	1	31	Cuidados Semi Intensivos

1	3	2	1	1	18	Cuidados Intermediários
1	3	2	1	1	19	Cuidados Intermediários
1	3	2	1	1	22	Cuidados Intermediários
1	3	2	1	1	20	Cuidados Intermediários
2	3	2	4	4	33	Cuidados Semi Intensivos
-	-	-	-	-	0	Vago
1	3	3	4	4	29	Alta Dependência
1	3	3	4	4	30	Alta Dependência
1	3	2	1	1	17	Cuidados Mínimos
1	4	2	2	2	34	Cuidados Semi Intensivos
1	3	2	1	1	24	Alta Dependência
4	4	2	4	4	34	Cuidados Semi Intensivos
1	3	2	2	2	25	Alta Dependência
-	-	-	-	-	0	Vago
1	3	3	4	4	30	Alta Dependência
-	-	-	-	-	0	Vago
1	3	2	1	1	21	Cuidados Intermediários
2	3	3	2	2	30	Alta Dependência
-	-	-	-	-	0	Vago
-	-	-	-	-	0	Vago
1	3	2	4	4	25	Alta Dependência
1	3	2	1	1	21	Cuidados Intermediários
2	3	2	4	4	33	Cuidados Semi Intensivos
-	-	-	-	-	0	Vago
2	3	3	4	4	31	Cuidados Semi Intensivos
1	3	3	4	4	30	Alta Dependência
2	3	2	1	1	18	Cuidados Intermediários
2	4	2	2	2	32	Cuidados Semi Intensivos
2	2	2	1	1	24	Alta Dependência
4	4	2	1	1	30	Alta Dependência
1	2	2	2	2	24	Alta Dependência
2	3	2	4	4	36	Cuidados Semi Intensivos
-	-	-	-	-	0	Vago
1	3	3	1	1	23	Cuidados Intermediários
1	3	2	1	1	18	Cuidados Intermediários
-	-	-	-	-	0	Vago
1	3	3	4	4	29	Alta Dependência
-	-	-	-	-	0	Vago
1	3	2	1	1	22	Cuidados Intermediários
1	3	2	4	4	24	Alta Dependência
1	3	2	4	4	28	Alta Dependência
-	-	-	-	-	0	Vago
1	3	3	4	4	28	Alta Dependência
1	3	3	4	4	28	Alta Dependência
1	3	2	4	4	22	Cuidados Intermediários
1	4	2	2	2	32	Cuidados Semi Intensivos
-	-	-	-	-	0	Vago
1	4	2	4	4	31	Cuidados Semi Intensivos
1	3	2	2	2	23	Cuidados Intermediários
1	3	2	4	4	31	Cuidados Semi Intensivos

-	-	-	-	-	0	Vago
1	3	3	4	4	25	Alta Dependência
1	3	2	4	4	24	Alta Dependência
1	3	2	4	4	28	Alta Dependência
1	3	3	4	4	25	Alta Dependência
-	-	-	-	-	0	Vago
1	3	2	4	4	20	Cuidados Intermediários
1	3	2	4	4	21	Cuidados Intermediários
1	3	2	4	4	26	Alta Dependência
1	3	2	4	4	21	Cuidados Intermediários
1	3	2	4	4	26	Alta Dependência
1	3	3	2	3	25	Alta Dependência
1	3	2	4	4	20	Cuidados Intermediários
1	3	3	2	3	33	Cuidados Semi Intensivos
1	3	2	4	4	24	Alta Dependência
4	3	2	4	4	31	Cuidados Semi Intensivos
1	3	2	2	2	21	Cuidados Intermediários
1	3	2	2	2	20	Cuidados Intermediários
-	-	-	-	-	0	Vago
1	3	3	4	4	24	Alta Dependência
1	3	2	4	4	23	Cuidados Intermediários
1	3	3	4	4	27	Alta Dependência
1	3	3	4	4	25	Alta Dependência
1	3	2	4	4	20	Cuidados Intermediários
1	3	3	4	4	21	Cuidados Intermediários

Dia	Dia_semana	Leito	Estado Mental e Atividade	Oxigenação	Mobilidade e Deambulação	Alimentação e Hidratação	Eliminações	Higiene e Cuidado Corporal
2	6	649A	3	1	4	2	3	4
2	6	649B	3	1	3	2	3	4
2	6	651A	-	-	-	-	-	-
2	6	651B	-	-	-	-	-	-
2	6	653A	2	1	3			3
2	6	653B	3	1	3	2	3	3
2	6	655A	-	-	-	-	-	-
2	6	655B	-	-	-	-	-	-
2	6	657A	3	2	4	3	3	4
2	6	657B	3	2	4	4	4	4
2	6	659A	2	1	3	2	3	3
2	6	659B	2	1	3	2	3	3
2	6	661A	2	1	1	1	1	2
2	6	661B	3	2	4	2	3	4
2	6	663A	2	1	2	1	1	2
2	6	663B	2	1	3	1	3	
2	6	663C	2	1	3	1	3	
2	6	663D	2	4	3	2	3	
3	7	649A	3	1	4	2	3	4
3	7	649B	3	1	4	2	4	4
3	7	651A	3	3	4	2	3	4
3	7	651B	1	1	1	2	2	2
3	7	653A	1	1	3	2	2	3
3	7	653B	1	1	3	2	2	3
3	7	655A	3	1	2	2	3	3
3	7	655B	4	4	4	4	4	4
3	7	657A	4	3	4	4	3	4
3	7	657B	4	3	4	4	4	4
3	7	659A	1	1	2	2	2	2
3	7	659B	1	1	2	2	2	2
3	7	661A	1	1	2	2	2	2
3	7	661B	3	1	4	3	3	4
3	7	663A	1	1	2	2	2	3
3	7	663B	1	1	2	2	2	3
3	7	663C	1	1	3	2	3	3
3	7	663D	1	3	2	2	3	3
4	1	649A	3	1	4	2	3	4
4	1	649B	3	1	4	2	4	4
4	1	651A	3	3	4	2	3	4
4	1	651B	1	1	1	2	1	2

4	1	653A	1	1	3	2	2	3
4	1	653B	1	1	3	2	2	3
4	1	655A	3	1	2	2	3	3
4	1	655B	4	4	4	3	4	4
4	1	657A	4	3	4	3	3	4
4	1	657B	4	4	4	4	4	4
4	1	659A	1	1	2	2	2	2
4	1	659B	1	1	2	2	2	2
4	1	661A	1	1	2	2	2	2
4	1	661B	3	1	4	3	3	4
4	1	663A	1	1	2	2	2	3
4	1	663B	1	1	2	2	2	3
4	1	663C	1	1	3	2	3	3
4	1	663D	1	3	2	2	3	3
5	2	649A	3	1	4	2	3	4
5	2	649B	3	1	4	2	3	4
5	2	651A	3	3	4	2	3	4
5	2	651B	1	1	1	2	1	2
5	2	653A	1	1	3	2	2	3
5	2	653B	1	1	3	2	2	3
5	2	655A	3	1	2	2	2	3
5	2	655B	4	4	4	3	4	4
5	2	657A	4	3	4	3	3	4
5	2	657B	4	4	4	3	4	4
5	2	659A	1	4	2	2	2	2
5	2	659B	-	-	-	-	-	-
5	2	661A	-	-	-	-	-	-
5	2	661B	3	3	4	4	4	4
5	2	663A	1	1	2	2	2	3
5	2	663B	1	1	2	2	2	3
5	2	663C	1	1	3	2	3	3
5	3	663D	1	3	2	2	3	3
6	3	649A	3	1	4	2	3	4
6	3	649B	3	1	4	2	3	4
6	3	651A	3	3	4	2	3	4
6	3	651B	1	1	1	2	1	2
6	3	653A	1	1	3	2	2	3
6	3	653B	1	1	3	2	2	3
6	3	655A	3	1	2	2	2	3
6	3	655B	4	4	4	3	4	4
6	3	657A	4	3	4	3	3	4
6	3	657B	4	4	4	3	4	4
6	3	659A	1	4	2	2	2	2
6	3	659B	1	1	2	2	2	2
6	3	661A	-	-	-	-	-	-
6	3	661B	3	3	4	4	4	4
6	3	663A	1	1	2	2	2	3

6	3	663B	1	1	2	2	2	3
6	3	663C	1	1	3	2	3	3
6	3	663D	1	3	2	2	3	3
7	4	649A	1	1	4	2	3	4
7	4	649B	1	1	4	2	3	
7	4	651A	1		3	2	3	3
7	4	651B	3	2	4	3	3	4
7	4	653A	1	1	3		3	3
7	4	653B	1	1	4		4	4
7	4	655A	-	-	-	-	-	-
7	4	655B	4	4	4	3	4	4
7	4	657A	3	3	4		3	4
7	4	657B	4	4	4		4	4
7	4	659A	1	3	4	3	4	4
7	4	659B	1	2	4	3	3	4
7	4	661A	2	3	4	3	4	4
7	4	661B	2	4	3	4	3	3
7	4	663A	1	1	1	2	2	3
7	4	663B	1	1	1	2	3	3
7	4	663C	1	1	2	2	3	3
7	4	663D	1	3	2	2	3	3
8	5	649A	1	1	4	2	3	4
8	5	649B	1	1	1	1	1	2
8	5	651A	-	-	-	-	-	-
8	5	651B	3	1	4	3	4	4
8	5	653A	1	1	4	2	3	4
8	5	653B	1	1	3	2	4	3
8	5	655A	1	1	4	2	4	4
8	5	655B	4	4	4	3	4	4
8	5	657A	3	3	4	3	4	4
8	5	657B	4	4	4	3	4	4
8	5	659A	1	1	3	2	4	4
8	5	659B	1	1	4	2	4	4
8	5	661A	1	3	4	2	4	4
8	5	661B	1	1	3	4	3	3
8	5	663A	1	1	3	2	2	3
8	5	663B	1	1	3	2	2	3
8	5	663C	1	1	3	1	2	3
8	5	663D	1	4	2	1	2	3
9	6	649A	2	1	4	2	3	4
9	6	649B	-	-	-	-	-	-
9	6	651A	-	-	-	-	-	-
9	6	651B	3	3	4	2	4	4
9	6	653A	1	1	3	1	3	3
9	6	653B	1	1	3	1	3	3
9	6	655A	1	1	4	2	4	4
9	6	655B	4	4	4	3	4	4

9	6	657A	4	3	4	3	3	4
9	6	657B	4	4	4	3	4	4
9	6	659A	1	1	4	2	4	4
9	6	659B	1	1	4	2	3	4
9	6	661A	1	3	4	2	4	4
9	6	661B	1	4	3	2	3	3
9	6	663A	3	1	3	2	3	3
9	6	663B	1	1	2	1	3	2
9	6	663C	1	1	3	1	3	3
9	6	663D	1	4	2	1	3	3
10	7	649A	1	1	2	1	3	3
10	7	649B	1	1	3	2	4	4
10	7	651A	-	-	-	-	-	-
10	7	651B	3	3	3	3	4	4
10	7	653A	1	1	3	1	3	4
10	7	653B	1	1	3	1	3	3
10	7	655A	1	1	3	1	4	4
10	7	655B	4	4	4	3	4	4
10	7	657A	-	-	-	-	-	-
10	7	657B	4	4	4	3	4	4
10	7	659A	1	1	3	1	4	4
10	7	659B	1	1	3	2	3	4
10	7	661A	1	3	3	3	4	4
10	7	661B	1	4	3	4	3	3
10	7	663A	3	1	3	2	3	3
10	7	663B	1	1	2	1	3	3
10	7	663C	1	1	3	1	3	4
10	7	663D	1	3	2	1	3	3
11	1	649A	3	1	3	2	3	3
11	1	649B	3	1	4	2	3	4
11	1	651A	-	-	-	-	-	-
11	1	651B	3	3	4	2	4	4
11	1	653A	3	1	4	2	3	4
11	1	653B	3	1	3	2	2	2
11	1	655A	3	1	4	3	3	4
11	1	655B	4	4	4	3	4	4
11	1	657A	-	-	-	-	-	-
11	1	657B	4	4	4	3	4	4
11	1	659A	3	3	4	2	3	4
11	1	659B	3	4	4	2	3	4
11	1	661A	3	3	4	2	4	4
11	1	661B	3	4	3	2	3	2
11	1	663A	3	1	4	2	3	2
11	1	663B	3	1	3	2	3	2
11	1	663C	1	1	3	2	2	2
11	1	663D	1	1	2	2	1	2
12	2	649A	2	2	2	1	3	3

12	2	649B	2	2	2	1	3	3
12	2	651A	-	-	-	-	-	-
12	2	651B	3	3	3	3	4	4
12	2	653A	3	2	3	1	3	4
12	2	653B	3	2	3	2	3	3
12	2	655A	3	2	4	3		4
12	2	655B	4	4	4	3	4	4
12	2	657A	3	3	4	3	3	4
12	2	657B	3	2	4	1	4	4
12	2	659A	3	2	3	2	3	4
12	2	659B	3	2	4	2	3	4
12	2	661A	3	3	4	3	4	4
12	2	661B	3	4	3	4	3	4
12	2	663A	3	2	3	2	3	4
12	2	663B	3	2	2	1	3	3
12	2	663C	3	2	2	1	1	2
12	3	663D	3	3	3	2	2	3
13	3	649A	1	1	2	1	3	3
13	3	649B	1	3	2	1	3	3
13	3	651A	-	-	-	-	-	-
13	3	651B	3	2	4	2	4	4
13	3	653A	1	1	3	3	3	4
13	3	653B	1	1	3	3	3	3
13	3	655A	1	3	3	2	4	4
13	3	655B	4	4	4	3	4	4
13	3	657A	3	3	4	3	4	4
13	3	657B	-	-	-	-	-	-
13	3	659A	-	-	-	-	-	-
13	3	659B	1	1	3	2	3	4
13	3	661A	1	2	4	2	4	4
13	3	661B	2	4	4	4	3	4
13	3	663A	-	-	-	-	-	-
13	3	663B	1	1	3	1	3	3
13	3	663C	1	1	2	1	3	3
13	3	663D	1	3	3	2	3	4
14	4	649A	1	1	3	1	3	3
14	4	649B	-	-	-	-	-	-
14	4	651A	-	-	-	-	-	-
14	4	651B	3	2	4	2	3	4
14	4	653A	1	1	3	1	3	3
14	4	653B	1	1	3	1	3	3
14	4	655A	1	1	3	2	4	4
14	4	655B	4	4	4	3	4	4
14	4	657A	4	4	4	3	4	4
14	4	657B	-	-	-	-	-	-
14	4	659A	3	3	3	2	4	4
14	4	659B	1	3	3	1	3	3

14	4	661A	1	2	3	2	4	4
14	4	661B	1	4	3	4	4	4
14	4	663A	1	1	3	2	4	3
14	4	663B	1	1	3	2	4	3
14	4	663C	1	1	3	2	4	3
14	4	663D	2	3	3	2	4	3
15	5	649A	1	1	3	1	3	3
15	5	649B	1	1	3	2	3	3
15	5	651A	1	1	3	2	3	4
15	5	651B	3	1	4	2	3	4
15	5	653A	3	2	4	2	3	4
15	5	653B	2	1	3	1	3	3
15	5	655A	3	3	4	2	3	4
15	5	655B	3	1	4	2	3	4
15	5	657A	-	-	-	-	-	-
15	5	657B	3	3	4	3	4	4
15	5	659A	3	1	4	2	3	4
15	5	659B	1	2	2	1	3	2
15	5	661A	1	3	4	3	4	4
15	5	661B	1	4	3	4	3	4
15	5	663A	1	1	2	2	2	2
15	5	663B	1	1	1	1	1	1
15	5	663C	1	1	2	1	2	2
15	5	663D	2	3	4	2	4	4
16	6	649A	1	1	3	1	3	3
16	6	649B	1	1	3	2	3	3
16	6	651A	1	1	3	2	3	4
16	6	651B	3	1	4	2	3	4
16	6	653A	3	2	4	2	3	4
16	6	653B	-	-	-	-	-	-
16	6	655A	3	3	4	2	3	4
16	6	655B	3	1	4	2	3	4
16	6	657A	-	-	-	-	-	-
16	6	657B	3	3	4	3	4	4
16	6	659A	3	1	4	2	3	4
16	6	659B	1	2	2	1	3	2
16	6	661A	1	3	4	3	3	4
16	6	661B	1	4	3	4	3	4
16	6	663A	1	1	2	2	2	2
16	6	663B	1	1	1	1	1	1
16	6	663C	1	1	2	1	2	2
16	6	663D	2	3	4	2	4	4
17	7	649A	1	1	2	1	2	3
17	7	649B	1	1	2	1	2	3
17	7	651A	1	1	2	1	2	3
17	7	651B	3	1	3	2	3	3
17	7	653A	3	1	4	2	3	3

17	7	653B	-	-	-	-	-	-
17	7	655A	1	1	3	1	2	2
17	7	655B	3	1	4	3	3	4
17	7	657A	1	3	3	1	3	3
17	7	657B	3	3	4	3	4	4
17	7	659A	-	-	-	-	-	-
17	7	659B	1	1	3	1	3	4
17	7	661A	1	3	4	1	4	4
17	7	661B	1	4	3	2	3	4
17	7	663A	1	1	4	1	4	4
17	7	663B	1	1	1	1	1	1
17	7	663C	1	1	1	1	1	1
17	7	663D	1	3	3	1	4	3
18	1	649A	1	1	4	2	3	4
18	1	649B	-	-	-	-	-	-
18	1	651A	1	1	3	2	3	3
18	1	651B	3	1	4	2	3	3
18	1	653A	3	1	4	2	3	4
18	1	653B	1	1	2	1	3	3
18	1	655A	1	2	3	2	3	3
18	1	655B	3	1	4	2	3	4
18	1	657A	3	1	3	2	3	3
18	1	657B	3	1	4	3	4	4
18	1	659A	1	3	4	2	3	4
18	1	659B	1	2	3	1	3	3
18	1	661A	1	3	4	2	4	4
18	1	661B	1	4	4	4	3	4
18	1	663A	1	1	3	2	1	3
18	1	663B	1	1	1	1	3	2
18	1	663C	-	-	-	-	-	-
18	1	663D	1	3	3	2	4	3
19	2	649A	1	1	4	2	3	4
19	2	649B	1	2	2	1	3	2
19	2	651A	1	1	3	2	3	3
19	2	651B	3	1	4	2	3	3
19	2	653A	3	1	4	2	3	4
19	2	653B	1	2	2	1	3	3
19	2	655A	1	1	3	2	3	3
19	2	655B	3	1	4	2	3	4
19	2	657A	3	1	3	2	3	3
19	2	657B	3	1	4	3	4	4
19	2	659A	1	3	4	2	3	4
19	2	659B	-	-	-	-	-	-
19	2	661A	1	2	4	2	4	4
19	2	661B	1	4	4	4	3	4
19	2	663A	-	-	-	-	-	-
19	2	663B	1	1	2	1	1	2

19	2	663C	1	1	4	2	3	3
19	3	663D	1	3	3	2	4	4
20	3	649A	1	1	4	2	3	4
20	3	649B	1	2	2	1	3	2
20	3	651A	1	1	3	2	4	3
20	3	651B	3	1	4	2	3	3
20	3	653A	3	1	4	2	3	4
20	3	653B	1	2	2	1	3	3
20	3	655A	1	1	3	2	3	3
20	3	655B	3	1	4	2	3	4
20	3	657A	3	1	3	2	3	3
20	3	657B	3	1	4	3	4	4
20	3	659A	1	4	4	2	3	4
20	3	659B	-	-	-	-	-	-
20	3	661A	1	2	4	2	4	4
20	3	661B	1	4	4	4	3	4
20	3	663A	1	1	2	2	3	3
20	3	663B	1	1	2	1	1	2
20	3	663C	-	-	-	-	-	-
20	3	663D	1	3	3	2	4	3
21	4	649A	1	1	4	2	3	4
21	4	649B	1	2	2	1	3	2
21	4	651A	1	1	3	2	4	3
21	4	651B	3	1	4	2	3	3
21	4	653A	3	1	4	2	3	4
21	4	653B	1	2	2	1	3	3
21	4	655A	1	1	3	2	3	3
21	4	655B	3	1	4	2	3	4
21	4	657A	3	1	3	2	3	3
21	4	657B	3	1	4	3	4	4
21	4	659A	1	4	4	2	3	4
21	4	659B	-	-	-	-	-	-
21	4	661A	1	2	4	2	4	4
21	4	661B	1	4	4	3	3	4
21	4	663A	1	1	2	2	3	3
21	4	663B	1	1	2	1	1	2
21	4	663C	-	-	-	-	-	-
21	4	663D	1	3	3	2	4	3
22	5	649A	2	1	2	1	1	2
22	5	649B	-	-	-	-	-	-
22	5	651A	3	1	3	2	3	3
22	5	651B	3	3	3	2	4	3
22	5	653A	3	2	4	3	4	4
22	5	653B	3	4	3	3	3	3
22	5	655A	4	3	4	4	4	4
22	5	655B	3	2	4	3	3	4
22	5	657A	2	2	2	2	1	2

22	5	657B	-	-	-	-	-	-
22	5	659A	3	2	3	2	3	3
22	5	659B	3	2	3	3	4	4
22	5	661A	3	2	3	3	3	4
22	5	661B	-	-	-	-	-	-
22	5	663A	-	-	-	-	-	-
22	5	663B	3	2	4	3	3	4
22	5	663C	3	4	4	3	3	4
22	5	663D	3	4	3	3	3	3
23	6	649A	3	3	4	2	3	
23	6	649B	1	1	2	1	2	
23	6	651A	1	1	2	2	2	
23	6	651B	1	1	2	1	2	
23	6	653A	3	1	4	2	2	
23	6	653B	-	-	-	-	-	-
23	6	655A	-	-	-	-	-	-
23	6	655B	3	1	3	2	4	
23	6	657A	-	-	-	-	-	-
23	6	657B	-	-	-	-	-	-
23	6	659A	-	-	-	-	-	-
23	6	659B	3	3	3	3	3	
23	6	661A	3	1	4	2	4	
23	6	661B	1	3	3	1	3	
23	6	663A	1	1	3	2	3	
23	6	663B	1	1	1	1	2	
23	6	663C	1	3	4	1	3	
23	6	663D	1	3	3	1	4	
24	7	649A	3	4	4	2	3	4
24	7	649B	3	3	2	1	3	2
24	7	651A	3	1	3	2	2	3
24	7	651B	3	1	3	2	2	3
24	7	653A	3	1	4	2	3	4
24	7	653B	-	-	-	-	-	-
24	7	655A	-	-	-	-	-	-
24	7	655B	3	1	4	2	3	4
24	7	657A		1	3	1	3	3
24	7	657B	3	1	3	1	3	3
24	7	659A	4	3	4	3	3	
24	7	659B	3	1	3	3	3	3
24	7	661A	3	3	4	2	4	4
24	7	661B	3	4	3	3	3	3
24	7	663A	2	1	2	2	2	2
24	7	663B	2	1	1	1	1	2
24	7	663C	3	3	3	2	3	3
24	7	663D	3	3	3	2	4	3
25	1	649A	3	4	4	2	3	4
25	1	649B	3	3	2	1	3	2

25	1	651A	3	1	3	2	2	3
25	1	651B	3	1	3	2	2	3
25	1	653A	3	1	4	2	3	4
25	1	653B	-	-	-	-	-	-
25	1	655A	-	-	-	-	-	-
25	1	655B	3	1	4	2	3	4
25	1	657A	2	1	3	1	3	3
25	1	657B	3	1	3	1	3	3
25	1	659A	4	3	4	3	3	
25	1	659B	3	1	3	3	3	3
25	1	661A	3	3	4	2	4	4
25	1	661B	3	4	3	3	3	3
25	1	663A	2	1	2	2	2	2
25	1	663B	2	1	1	1	1	2
25	1	663C	3	3	3	2	3	3
25	1	663D	3	3	3	2	4	3
26	2	649A	3	4	3	3	3	4
26	2	649B	3	4	3	2	3	3
26	2	651A	3	1	3	4	3	3
26	2	651B	3	1	3	4	3	3
26	2	653A	3	1	4	3	3	4
26	2	653B	-	-	-	-	-	-
26	2	655A	2	1	2	1	1	2
26	2	655B	3	1	4	3	4	4
26	2	657A	2	1	2	2	3	3
26	2	657B	3	1	4	2	3	4
26	2	659A	4	3	4	3		4
26	2	659B	3	1	3	2		3
26	2	661A	3	2	4	3	3	4
26	2	661B	3	4	3	3	3	3
26	2	663A	2	1	3	1	3	3
26	2	663B	2	1	3	1	3	3
26	2	663C	3	3	3	2	3	3
26	3	663D	3	3	3	2	3	3
27	3	649A	3	4	3	3	3	4
27	3	649B	3	4	3	2	3	3
27	3	651A	3	1	3	4	3	3
27	3	651B	3	1	3	4	3	3
27	3	653A	3	1	4	3	3	4
27	3	653B	-	-	-	-	-	-
27	3	655A	2	1	2	1	1	2
27	3	655B	3	1	4	3	4	4
27	3	657A	2	1	2	2	3	3
27	3	657B	3	1	4	2	3	4
27	3	659A	-	-	-	-	-	-
27	3	659B	3	1	3	2	2	3
27	3	661A	-	-	-	-	-	-

27	3	661B	3	4	3	3	3	3
27	3	663A	2	1	3	1	3	3
27	3	663B	3	1	3	1	3	4
27	3	663C	3	3	3	2	3	3
27	3	663D	3	3	3	2	3	3
28	4	649A	3	4	4	2	3	4
28	4	649B	3	4	3	1	3	3
28	4	651A	3	1	2	1	2	2
28	4	651B	-	-	-	-	-	-
28	4	653A	3	3	4	2	3	4
28	4	653B	-	-	-	-	-	-
28	4	655A	-	-	-	-	-	-
28	4	655B	3	2	4	2	3	4
28	4	657A	3	1	3	1	2	3
28	4	657B	3	1	4	2	3	4
28	4	659A	-	-	-	-	-	-
28	4	659B	3	2	3	3	3	3
28	4	661A	-	-	-	-	-	-
28	4	661B	3	4	3	2	3	3
28	4	663A	3	1	2		2	3
28	4	663B	3	2	4	3	3	4
28	4	663C	3	3	3	2	3	3
28	4	663D	3	3	3	2	3	3
29	5	649A	3	4	4	2	3	4
29	5	649B	3	4	3	1	3	3
29	5	651A	3	1	3	1	2	3
29	5	651B	-	-	-	-	-	-
29	5	653A	-	-	-	-	-	-
29	5	653B	-	-	-	-	-	-
29	5	655A	3	2	4	3	3	4
29	5	655B	3	3	4	3	3	4
29	5	657A	3	1	3	2	2	3
29	5	657B	3	1	4	2	3	4
29	5	659A	-	-	-	-	-	-
29	5	659B	3	2	3	3	3	3
29	5	661A	3	2	3	2	3	3
29	5	661B	3	2	4	2	3	4
29	5	663A	-	-	-	-	-	-
29	5	663B	3	1	3	1	2	3
29	5	663C	3	3	3	2	2	3
29	5	663D	3	3	3	2	4	3
30	6	649A	3	4	4	2	3	4
30	6	649B	3	4	3	1	3	3
30	6	651A	3	1	3	1	2	3
30	6	651B	-	-	-	-	-	-
30	6	653A	3	1	2			2
30	6	653B	3	1	2			2

30	6	655A	3	2	4	3	3	4
30	6	655B	3	3	4	3	3	4
30	6	657A	3	1	3	2	2	3
30	6	657B	3	1	4	2	3	4
30	6	659A	-	-	-	-	-	-
30	6	659B	3	2	3	3	3	3
30	6	661A	3	2	3	2	3	3
30	6	661B	3	2	4	2	3	4
30	6	663A	-	-	-	-	-	-
30	6	663B	3	1	3	1	2	3
30	6	663C	3	3	3	2	2	3
30	6	663D	3	3	3	2	4	3

Intervalo de Aferição de Controles	Terapêutica Medicamentosa	Integridade Cutâneo Mucosa	Participação do acompanhante	Rede de apoio e suporte	TOTAL	Categoria de Cuidado
1	3	3	4	4	32	Cuidados Semi Intensivos
1	3	2	2	2	26	Alta Dependência
-	-	-	-	-	0	Vago
-	-	-	-	-	0	Vago
1	3	4	3	1	21	Cuidados Intermediários
1	3	3	3	3	28	Alta Dependência
-	-	-	-	-	0	Vago
-	-	-	-	-	0	Vago
4	4	3	4	4	38	Cuidados Intensivos
4	4	3	4	4	40	Cuidados Intensivos
4	4	2	4	4	32	Cuidados Semi Intensivos
1	3	2	2	3	25	Alta Dependência
1	3	2	1	1	16	Cuidados Mínimos
1	3	3	3	3	31	Cuidados Semi Intensivos
1	3	2	3	3	21	Cuidados Intermediários
1	3	2	2	1	19	Cuidados Intermediários
1	3	4	1	1	20	Cuidados Intermediários
1	3	2	1	1	22	Cuidados Intermediários
1	3	3	4	4	32	Cuidados Semi Intensivos
1	3	2	2	2	28	Alta Dependência
1	3	2	4	4	33	Cuidados Semi Intensivos
	3	2	2	1	17	Cuidados Mínimos
1	3	3	2	1	22	Cuidados Intermediários
1	3	3	2	1	22	Cuidados Intermediários
1	3	2	2	4	26	Alta Dependência
4	4	4	4	4	44	Cuidados Intensivos
4	3	4	4	4	41	Cuidados Intensivos
4	4	4	4	4	43	Cuidados Intensivos
4	4	2	4	4	28	Alta Dependência
1	3	2	4	4	24	Alta Dependência
1	3	2	4	4	24	Alta Dependência
1	3	3	2	2	29	Alta Dependência
1	3	2	4	4	25	Alta Dependência
1	3	2	4	4	25	Alta Dependência
1	3	3	4	4	28	Alta Dependência
1	3	2	4	4	28	Alta Dependência
1	3	3	4	4	32	Cuidados Semi Intensivos
1	3	2	2	3	29	Alta Dependência
1	3	2	4	4	33	Cuidados Semi Intensivos
1	3	2	2	1	17	Cuidados Mínimos

1	3	3	2	3	24	Alta Dependência
1	3	3	2	3	24	Alta Dependência
1	3	2	2	4	26	Alta Dependência
4	4	4	4	4	43	Cuidados Intensivos
4	3	4	4	4	40	Cuidados Intensivos
4	4	4	4	4	44	Cuidados Intensivos
4	4	2	4	4	28	Alta Dependência
1	3	2	4	4	24	Alta Dependência
1	3	2	4	4	24	Alta Dependência
1	3	3	2	3	30	Alta Dependência
1	3	2	4	4	25	Alta Dependência
1	3	2	4	4	25	Alta Dependência
1	3	3	4	4	28	Alta Dependência
1	3	2	4	4	28	Alta Dependência
1	3	3	4	4	32	Cuidados Semi Intensivos
1	3	2	2	3	28	Alta Dependência
1	3	2	4	4	33	Cuidados Semi Intensivos
1	3	2	2	1	17	Cuidados Mínimos
1	3	3	2	3	24	Alta Dependência
1	3	3	2	3	24	Alta Dependência
1	3	2	2	4	25	Alta Dependência
4	4	4	4	4	43	Cuidados Intensivos
4	3	4	4	4	40	Cuidados Intensivos
4	4	4	4	4	43	Cuidados Intensivos
4	4	2		4	27	Alta Dependência
-	-	-	-	-	0	Vago
-	-	-	-	-	0	Vago
4	4	3	2	2	37	Cuidados Intensivos
1	3	2	4	4	25	Alta Dependência
1	3	2	4	4	25	Alta Dependência
1	3	3	4	4	28	Alta Dependência
1	3	2	4	4	28	Alta Dependência
1	3	3	4	4	32	Cuidados Semi Intensivos
1	3	2	2	3	28	Alta Dependência
1	3	2	4	4	33	Cuidados Semi Intensivos
1	3	2	2	1	17	Cuidados Mínimos
1	3	3	2	3	24	Alta Dependência
1	3	3	2	3	24	Alta Dependência
1	3	2	2	4	25	Alta Dependência
4	4	4	4	4	43	Cuidados Intensivos
4	3	4	4	4	40	Cuidados Intensivos
4	4	4	4	4	43	Cuidados Intensivos
4	4	2		4	27	Alta Dependência
1	3	2	4	4	24	Alta Dependência
-	-	-	-	-	0	Vago
4	4	3	2	2	37	Cuidados Intensivos
1	3	2	4	4	25	Alta Dependência

1	3	2	4	4	25	Alta Dependência
1	3	3	4	4	28	Alta Dependência
1	3	2	4	4	28	Alta Dependência
1	3	3	4	4	30	Alta Dependência
1	3	2	4	4	25	Alta Dependência
1	3	2	4	4	26	Alta Dependência
4	3	3	3	4	36	Cuidados Semi Intensivos
1	3	4	4	4	27	Alta Dependência
1	3	3	2	3	1	Vago
-	-	-	-	-	0	Vago
4	4	4	4	4	43	Cuidados Intensivos
4	3	3	4	4	35	Cuidados Semi Intensivos
4	4	4	4	4	40	Cuidados Intensivos
4	4	3	3	3	36	Cuidados Semi Intensivos
4	3	3	3	3	33	Cuidados Semi Intensivos
4	3	3	4	4	38	Cuidados Intensivos
1	4	3	2	2	31	Cuidados Semi Intensivos
1	3	2	4	4	24	Alta Dependência
1	3	2	4	4	25	Alta Dependência
1	3	3	4	4	27	Alta Dependência
1	3	2	4	4	28	Alta Dependência
1	3	2	4	4	29	Alta Dependência
1	3	2	4	4	21	Cuidados Intermediários
-	-	-	-	-	0	Vago
4	4	3	2	3	35	Cuidados Semi Intensivos
1	2	3	4	4	29	Alta Dependência
1	4	4	2	2	27	Alta Dependência
4	2	3	4	4	33	Cuidados Semi Intensivos
4	4	3	4	4	42	Cuidados Intensivos
4	3	3	4	4	39	Cuidados Intensivos
4	4	4	4	4	43	Cuidados Intensivos
4	2	3	2	2	28	Alta Dependência
4	2	3	2	2	29	Alta Dependência
1	2	3	2	2	28	Alta Dependência
1	4	3	2	2	27	Alta Dependência
1	2	3	2	2	22	Cuidados Intermediários
1	2	2	2	2	21	Cuidados Intermediários
1	2	3	2	2	21	Cuidados Intermediários
1	2	2	4	4	26	Alta Dependência
1	3	3	4	4	31	Cuidados Semi Intensivos
-	-	-	-	-	0	Vago
-	-	-	-	-	0	Vago
4	4	3	2	3	36	Cuidados Semi Intensivos
1	3	4	4	4	28	Alta Dependência
1	3	3	2	2	23	Cuidados Intermediários
4	3	3	4	4	34	Cuidados Semi Intensivos
4	4	3	4	4	42	Cuidados Intensivos

4	3	3	4	4	39	Cuidados Intensivos
4	4	3	4	4	42	Cuidados Intensivos
1	3	3	2	3	28	Alta Dependência
1	3	3	2	3	27	Alta Dependência
1	3	3	2	3	30	Alta Dependência
1	4	3	2	3	29	Alta Dependência
1	3	2	2	3	26	Alta Dependência
1	3	2	4	4	24	Alta Dependência
1	3	3	4	4	27	Alta Dependência
1	3	2	4	4	28	Alta Dependência
	3	2	4	4	24	Alta Dependência
	3	3	4	4	29	Alta Dependência
-	-	-	-	-	0	Vago
4	4	3	2	3	36	Cuidados Semi Intensivos
1	4	4	4	4	30	Alta Dependência
1	3	3	2	3	24	Alta Dependência
1	3	3	4	4	29	Alta Dependência
4	4	4	4	4	43	Cuidados Intensivos
-	-	-	-	-	0	Vago
4	4	4	4	4	43	Cuidados Intensivos
4	3	3	2	3	29	Alta Dependência
4	3	3	2	2	28	Alta Dependência
1	3	3	2	3	30	Alta Dependência
1	4	3	2	2	30	Alta Dependência
1	3	2	2	2	25	Alta Dependência
1	3	2	4	3	24	Alta Dependência
4	3	3	4	3	30	Alta Dependência
1	3	2	4	3	26	Alta Dependência
1	3	3	4	4	30	Alta Dependência
1	3	3	4	4	32	Cuidados Semi Intensivos
-	-	-	-	-	0	Vago
2	4	3	2	3	34	Cuidados Semi Intensivos
1	3	3	4	4	32	Cuidados Semi Intensivos
1	3	4	2	2	25	Alta Dependência
1	3	2	4	4	32	Cuidados Semi Intensivos
1	4	3	4	4	39	Cuidados Intensivos
-	-	-	-	-	0	Vago
2	4	4	4	4	41	Cuidados Intensivos
2	3	2	2	2	30	Alta Dependência
2	3	2	3	3	33	Cuidados Semi Intensivos
1	3	3	2	2	31	Cuidados Semi Intensivos
2	3	3	1	1	27	Alta Dependência
1	3	4	2	2	27	Alta Dependência
1	3	3	2	2	25	Alta Dependência
1	3	2	4	1	22	Cuidados Intermediários
1	3	2	4	1	20	Cuidados Intermediários
1	3	3	4	4	28	Alta Dependência

1	3	2	1	1	21	Cuidados Intermediários
-	-	-	-	-	0	Vago
2	3	3	2	2	32	Cuidados Semi Intensivos
1	3	3	1	1	25	Alta Dependência
2	3	3	2	2	28	Alta Dependência
2	3	3	4	4	32	Cuidados Semi Intensivos
2	4	3	4	4	40	Cuidados Intensivos
4	3	3	1	1	32	Cuidados Semi Intensivos
1	3	3	2	2	29	Alta Dependência
2	3	2	4	4	32	Cuidados Semi Intensivos
4	3	3	4	4	36	Cuidados Semi Intensivos
3	3	3	2	2	34	Cuidados Semi Intensivos
2	3	3	2	2	33	Cuidados Semi Intensivos
2	3	3	2	2	29	Alta Dependência
1	3	2	2	2	24	Alta Dependência
1	3	2	1	1	19	Cuidados Intermediários
1	3	2	4	4	30	Alta Dependência
1	3	3	4	4	26	Alta Dependência
1	3	2	4	4	27	Alta Dependência
-	-	-	-	-	0	Vago
4	3	3	2	3	34	Cuidados Semi Intensivos
1	3	4	4	4	31	Cuidados Semi Intensivos
1	3	3	2	3	26	Alta Dependência
4	3	3	4	4	35	Cuidados Semi Intensivos
4	4	4	4	4	43	Cuidados Intensivos
4	4	3	2	2	36	Cuidados Semi Intensivos
-	-	-	-	-	0	Vago
-	-	-	-	-	0	Vago
4	3	3	2	3	29	Alta Dependência
4	3	3	2	3	32	Cuidados Semi Intensivos
4	4	3	2	3	37	Cuidados Intensivos
-	-	-	-	-	0	Vago
1	3	3	4	3	26	Alta Dependência
1	3	3	4	3	25	Alta Dependência
1	3	2	3	3	28	Alta Dependência
1	3	3	4	4	27	Alta Dependência
-	-	-	-	-	0	Vago
-	-	-	-	-	0	Vago
4	3	3	4	4	36	Cuidados Semi Intensivos
	3	4	4	4	27	Alta Dependência
	3	3	4	4	26	Alta Dependência
	3	2	2	3	25	Alta Dependência
	4	4	4	4	39	Cuidados Intensivos
4	4	4	4	4	43	Cuidados Intensivos
-	-	-	-	-	0	Vago
	3	2	4	4	32	Cuidados Semi Intensivos
	3	2	4	4	27	Alta Dependência

4	3	3	2	3	31	Cuidados Semi Intensivos
4	4	3	2	3	36	Cuidados Semi Intensivos
1	3	3	4	4	29	Alta Dependência
1	3	3	4	4	29	Alta Dependência
1	3	3	4	4	29	Alta Dependência
1	3	3	4	4	32	Cuidados Semi Intensivos
1	3	3	4	4	27	Alta Dependência
1	3	3	2	1	23	Cuidados Intermediários
1	3	2	2	1	23	Cuidados Intermediários
1	3	2	2	1	26	Alta Dependência
2	3	3	2	1	29	Alta Dependência
1	3	3	2	1	23	Cuidados Intermediários
4	3	3	4	4	37	Cuidados Intensivos
1	3	2	3	2	28	Alta Dependência
-	-	-	-	-	0	Vago
1	3	3	4	4	36	Cuidados Semi Intensivos
4	3	2	2	1	29	Alta Dependência
4	4	2	4	4	29	Alta Dependência
4	3	3	2	1	32	Cuidados Semi Intensivos
4	4	3	2	1	33	Cuidados Semi Intensivos
1	3	3	2	1	20	Cuidados Intermediários
1	3	2	1	1	14	Cuidados Mínimos
1	3	2	2	2	19	Cuidados Intermediários
1	3	2	2	2	29	Alta Dependência
1	3	3	4	4	27	Alta Dependência
1	3	3	2	1	23	Cuidados Intermediários
1	3	2	2	1	23	Cuidados Intermediários
1	3	2	2	1	26	Alta Dependência
2	3	3	2	1	29	Alta Dependência
-	-	-	-	-	0	Vago
4	3	3	4	4	37	Cuidados Intensivos
1	3	2	3	2	28	Alta Dependência
-	-	-	-	-	0	Vago
1	3	3	4	4	36	Cuidados Semi Intensivos
4	3	2	2	1	29	Alta Dependência
4	4	2	4	4	29	Alta Dependência
4	3	3	2	1	31	Cuidados Semi Intensivos
4	4	3	2	1	33	Cuidados Semi Intensivos
1	3	3	2	1	20	Cuidados Intermediários
1	3	2	1	1	14	Cuidados Mínimos
1	3	2	2	2	19	Cuidados Intermediários
1	3	2	2	2	29	Alta Dependência
1	3	3	4	4	25	Alta Dependência
1	3	3	4	4	25	Alta Dependência
1	3	2	2	3	21	Cuidados Intermediários
1	3	2	2	1	24	Alta Dependência
1	3	2	3	2	27	Alta Dependência

-	-	-	-	-	0	Vago
1	3	2	2	2	20	Cuidados Intermediários
1	3	2	3	4	31	Cuidados Semi Intensivos
1	3	3	4	4	29	Alta Dependência
2	3	3	4	4	37	Cuidados Intensivos
-	-	-	-	-	0	Vago
2	4	2	1	4	26	Alta Dependência
1	3	3	2	1	27	Alta Dependência
1	4	3	4	1	30	Alta Dependência
1	3	3	1	1	24	Alta Dependência
1	3	2	2	3	17	Cuidados Mínimos
1	3	2	2	3	17	Cuidados Mínimos
1	3	2	3	4	28	Alta Dependência
1	3	3	4	4	30	Alta Dependência
-	-	-	-	-	0	Vago
1	3	2	2	1	22	Cuidados Intermediários
1	3	2	2	1	25	Alta Dependência
1	3	3	2	1	27	Alta Dependência
1	3	3	2	1	21	Cuidados Intermediários
4	3	2	4	4	31	Cuidados Semi Intensivos
1	3	3	3	2	29	Alta Dependência
1	3	3	1	1	24	Alta Dependência
1	3	3	4	4	34	Cuidados Semi Intensivos
1	3	2	4	1	28	Alta Dependência
4	4	2	4	4	31	Cuidados Semi Intensivos
1	3	3	2	1	28	Alta Dependência
4	4	3	2	1	34	Cuidados Semi Intensivos
1	3	2	2	1	20	Cuidados Intermediários
1	3	2	1	1	17	Cuidados Mínimos
-	-	-	-	-	0	Vago
1	3	2	2	2	26	Alta Dependência
1	3	3	4	4	30	Alta Dependência
2	4	2	2	1	22	Cuidados Intermediários
1	3	2	2	1	22	Cuidados Intermediários
1	3	2	2	1	25	Alta Dependência
1	3	3	2	1	27	Alta Dependência
1	3	3	2	1	22	Cuidados Intermediários
4	3	2	2	2	26	Alta Dependência
1	3	3	3	2	29	Alta Dependência
1	3	3	4	1	27	Alta Dependência
1	3	3	4	4	34	Cuidados Semi Intensivos
1	3	2	4	4	31	Cuidados Semi Intensivos
-	-	-	-	-	0	Vago
1	3	3	2	2	28	Alta Dependência
4	4	3	2	2	35	Cuidados Semi Intensivos
-	-	-	-	-	0	Vago
1	3	2	1	1	16	Cuidados Mínimos

1	3	3	2	2	25	Alta Dependência
1	3	2	2	2	27	Alta Dependência
1	3	3	4	4	30	Alta Dependência
2	4	2	2	1	22	Cuidados Intermediários
1	3	2	2	1	23	Cuidados Intermediários
1	3	2	2	1	25	Alta Dependência
4	3	3	2	1	30	Alta Dependência
1	3	3	2	1	22	Cuidados Intermediários
4	3	2	2	2	26	Alta Dependência
1	3	3	3	2	29	Alta Dependência
1	3	3	4	1	27	Alta Dependência
1	3	3	4	4	34	Cuidados Semi Intensivos
1	3	2	4	4	32	Cuidados Semi Intensivos
-	-	-	-	-	0	Vago
1	3	3	2	1	27	Alta Dependência
4	4	3	2	1	34	Cuidados Semi Intensivos
1	3	2	1	1	20	Cuidados Intermediários
1	3	2	1	1	16	Cuidados Mínimos
-	-	-	-	-	0	Vago
1	3	2	2	2	26	Alta Dependência
1	3	3	4	4	30	Alta Dependência
3	4	2	2	1	23	Cuidados Intermediários
1	3	2	2	1	23	Cuidados Intermediários
1	3	2	2	1	25	Alta Dependência
4	3	3	2	1	30	Alta Dependência
1	3	3	2	1	22	Cuidados Intermediários
	3	2	2	2	22	Cuidados Intermediários
1	3	3	3	2	29	Alta Dependência
1	3	3	4	1	27	Alta Dependência
1	3	3	4	4	34	Cuidados Semi Intensivos
1	3	2	4	4	32	Cuidados Semi Intensivos
-	-	-	-	-	0	Vago
1	3	3	2	1	27	Alta Dependência
4	4	3	2	1	33	Cuidados Semi Intensivos
1	3	2	1	1	20	Cuidados Intermediários
1	3	2	1	1	16	Cuidados Mínimos
-	-	-	-	-	0	Vago
1	3	2	2	2	26	Alta Dependência
1	3	2	4	4	23	Cuidados Intermediários
-	-	-	-	-	0	Vago
1	3	3	4	4	30	Alta Dependência
2	3	2	4	4	33	Cuidados Semi Intensivos
2	3	3	2	2	32	Cuidados Semi Intensivos
2	3	3	2	2	31	Cuidados Semi Intensivos
4	4	3	2	2	38	Cuidados Intensivos
2	3	3	1	1	29	Alta Dependência
1	3	2	1	1	19	Cuidados Intermediários

-	-	-	-	-	0	Vago
1	3	2	4	4	30	Alta Dependência
1	3	2	4	4	33	Cuidados Semi Intensivos
2	3	3	1	1	28	Alta Dependência
-	-	-	-	-	0	Vago
-	-	-	-	-	0	Vago
2	3	2	2	2	30	Alta Dependência
4	3	2	2	2	34	Cuidados Semi Intensivos
4	4	2	1	1	31	Cuidados Semi Intensivos
			2	2	19	Cuidados Intermediários
			2		9	Vago
			4		12	Cuidados Mínimos
			1		8	Vago
			2		14	Cuidados Mínimos
-	-	-	-	-	0	Vago
-	-	-	-	-	0	Vago
			3	4	20	Cuidados Intermediários
-	-	-	-	-	0	Vago
-	-	-	-	-	0	Vago
-	-	-	-	-	0	Vago
			1		16	Cuidados Mínimos
			2		16	Cuidados Mínimos
			3	2	16	Cuidados Mínimos
			2	3	15	Cuidados Mínimos
			4	3	13	Cuidados Mínimos
			1	1	14	Cuidados Mínimos
			3	3	18	Cuidados Intermediários
1	3	3	4	4	35	Cuidados Semi Intensivos
4	4	2	2	1	27	Alta Dependência
1	3	3	4	4	29	Alta Dependência
1	3	3	4	4	29	Alta Dependência
4	3	3	2	3	32	Cuidados Semi Intensivos
-	-	-	-	-	0	Vago
-	-	-	-	-	0	Vago
1	3	3	3	4	31	Cuidados Semi Intensivos
1	3	3	1	4	23	Cuidados Intermediários
1	3	3	1	4	26	Alta Dependência
					17	Cuidados Mínimos
1	3	3	4	4	31	Cuidados Semi Intensivos
1	3	3	2	2	31	Cuidados Semi Intensivos
1	3	3	2	2	30	Alta Dependência
1	3	3	1	1	20	Cuidados Intermediários
1	3	2	1	1	16	Cuidados Mínimos
1	3	2	4	4	31	Cuidados Semi Intensivos
1	3	2	2	2	28	Alta Dependência
1	3	3	4	4	35	Cuidados Semi Intensivos
4	4	2	2	1	27	Alta Dependência

1	3	3	4	4	29	Alta Dependência
1	3	3	4	4	29	Alta Dependência
4	3	3	2	3	32	Cuidados Semi Intensivos
-	-	-	-	-	0	Vago
-	-	-	-	-	0	Vago
1	3	3	3	4	31	Cuidados Semi Intensivos
1	3	3	1	4	25	Alta Dependência
1	3	3	1	4	26	Alta Dependência
					17	Cuidados Mínimos
1	3	3	4	4	31	Cuidados Semi Intensivos
1	3	3	2	2	31	Cuidados Semi Intensivos
1	3	3	2	2	30	Alta Dependência
1	3	3	1	1	20	Cuidados Intermediários
1	3	2	1	1	16	Cuidados Mínimos
1	3	2	4	4	31	Cuidados Semi Intensivos
1	3	2	2	2	28	Alta Dependência
2	3	2	4	4	35	Cuidados Semi Intensivos
4	4	2	2	2	32	Cuidados Semi Intensivos
1	3	3	4	4	32	Cuidados Semi Intensivos
1	3	3	4	4	32	Cuidados Semi Intensivos
2	3	2	1	1	27	Alta Dependência
-	-	-	-	-	0	Vago
1	3	2	1	1	17	Cuidados Mínimos
1	3	2	4	4	33	Cuidados Semi Intensivos
1	3	3	1	1	22	Cuidados Intermediários
1	3	3	1	1	26	Alta Dependência
1	3	4	1	1	28	Alta Dependência
1	3	2	1	1	20	Cuidados Intermediários
1	3	3	2	2	30	Alta Dependência
1	3	3	2	2	30	Alta Dependência
1	3	3	4	4	28	Alta Dependência
1	3	2	4	4	27	Alta Dependência
2	3	3	4	4	33	Cuidados Semi Intensivos
2	3	2	4	4	32	Cuidados Semi Intensivos
2	3	2	4	4	35	Cuidados Semi Intensivos
4	4	2	1	2	31	Cuidados Semi Intensivos
1	3	3	4	4	32	Cuidados Semi Intensivos
1	3	3	4	4	32	Cuidados Semi Intensivos
2	3	2	2	2	29	Alta Dependência
-	-	-	-	-	0	Vago
1	3	2	1	1	17	Cuidados Mínimos
1	3	2	4	4	33	Cuidados Semi Intensivos
1	3	3	4	4	28	Alta Dependência
1	3	3	4	4	32	Cuidados Semi Intensivos
-	-	-	-	-	0	Vago
1	3	2	4	3	27	Alta Dependência
-	-	-	-	-	0	Vago

1	3	3	2	2	30	Alta Dependência
1	3	3	2	3	25	Alta Dependência
1	3	2	2	3	26	Alta Dependência
1	3	3	4	4	32	Cuidados Semi Intensivos
1	3	2	4	3	30	Alta Dependência
2	2	2	4	3	33	Cuidados Semi Intensivos
2	4	2	2	2	29	Alta Dependência
1	2	3	4	4	25	Alta Dependência
-	-	-	-	-	0	Vago
1	2	3	2	2	29	Alta Dependência
-	-	-	-	-	0	Vago
-	-	-	-	-	0	Vago
1	3	3	3	4	32	Cuidados Semi Intensivos
1	2	3	1	1	21	Cuidados Intermediários
2	2	3	4	4	32	Cuidados Semi Intensivos
-	-	-	-	-	0	Vago
1	3	3	4	4	32	Cuidados Semi Intensivos
-	-	-	-	-	0	Vago
1	2	3	2	2	28	Alta Dependência
1	2	3	2	2	21	Cuidados Intermediários
1	3	2	2	2	29	Alta Dependência
1	2	3	4	4	31	Cuidados Semi Intensivos
1	2	2	2	2	26	Alta Dependência
2	2	2	4	3	33	Cuidados Semi Intensivos
2	4	2	2	2	29	Alta Dependência
1	2	3	4	4	27	Alta Dependência
-	-	-	-	-	0	Vago
-	-	-	-	-	0	Vago
-	-	-	-	-	0	Vago
1	3	3	2	2	30	Alta Dependência
1	3	3	2	2	31	Cuidados Semi Intensivos
1	2	3	1	1	22	Cuidados Intermediários
1	2	3	4	4	31	Cuidados Semi Intensivos
-	-	-	-	-	0	Vago
1	3	3	4	4	32	Cuidados Semi Intensivos
1	2	3	2	2	26	Alta Dependência
1	2	3	2	2	28	Alta Dependência
-	-	-	-	-	0	Vago
1	2	3	1	1	21	Cuidados Intermediários
1	2	3	4	4	30	Alta Dependência
1	2	2	2	3	28	Alta Dependência
2	2	2	4	3	33	Cuidados Semi Intensivos
2	4	2	2	2	29	Alta Dependência
1	2	3	4	4	27	Alta Dependência
-	-	-	-	-	0	Vago
1	2	2	1	1	15	Cuidados Mínimos
1	2	2	1	1	15	Cuidados Mínimos

1	3	3	2	2	30	Alta Dependência
1	3	3	2	2	31	Cuidados Semi Intensivos
1	2	3	1	1	22	Cuidados Intermediários
1	2	3	4	4	31	Cuidados Semi Intensivos
-	-	-	-	-	0	Vago
1	3	3	4	4	32	Cuidados Semi Intensivos
1	2	3	2	2	26	Alta Dependência
1	2	3	2	2	28	Alta Dependência
-	-	-	-	-	0	Vago
1	2	3	1	1	21	Cuidados Intermediários
1	2	3	4	4	30	Alta Dependência
1	2	2	2	3	28	Alta Dependência
