# Supplementary material for: Adaptation and validation of an adult patient classification instrument with emphasis on the family dimension
Source: Rev Bras Enferm. 2023 Mar 27;76(2):e20220530. doi: 10.1590/0034-7167-2022-0530 (PMC10042477; doi:10.1590/0034-7167-2022-0530)
Supplement: Supplementary file 6 [file 0034-7167-reben-76-02-e20220530-suppl06.pdf]

Dia do mês	Mínimos		Intermediários		Alta Dependência		Semi-Intensivos		Intensiv
	n	%	n	%	n	%	n	%	n
2	0	0.00	14	50.00	11	39.29	3	10.71	0
3	0	0.00	18	52.94	12	35.29	3	8.82	1
4	0	0.00	16	50.00	10	31.25	3	9.38	3
5	0	0.00	18	52.94	9	26.47	3	8.82	4
6	0	0.00	18	54.55	7	21.21	4	12.12	4
7	0	0.00	16	48.48	11	33.33	2	6.06	4
8	0	0.00	15	46.88	10	31.25	3	9.38	4
9	0	0.00	17	54.84	6	19.35	4	12.90	4
10	0	0.00	12	36.36	12	36.36	5	15.15	4
11	0	0.00	10	33.33	12	40.00	5	16.67	3
12	0	0.00	10	31.25	13	40.63	6	18.75	3
13	1	3.23	11	35.48	12	38.71	4	12.90	3
14	0	0.00	11	34.38	10	31.25	7	21.88	4
15	0	0.00	7	24.14	13	44.83	6	20.69	3
16	0	0.00	8	25.00	15	46.88	6	18.75	3
17	0	0.00	9	28.13	15	46.88	6	18.75	2
18	0	0.00	9	30.00	13	43.33	6	20.00	2
19	1	3.23	6	19.35	17	54.84	5	16.13	2
20	0	0.00	9	30.00	12	40.00	7	23.33	2
21	0	0.00	7	21.88	16	50.00	6	18.75	3
22	1	3.45	6	20.69	14	48.28	5	17.24	3
23	1	3.13	12	37.50	12	37.50	4	12.50	3
24	0	0.00	12	37.50	11	34.38	6	18.75	3
25	0	0.00	12	37.50	11	34.38	6	18.75	3
26	0	0.00	16	51.61	12	38.71	1	3.23	2
27	0	0.00	18	54.55	12	36.36	1	3.03	2
28	0	0.00	16	53.33	11	36.67	1	3.33	2
29	0	0.00	16	51.61	11	35.48	2	6.45	2
30	0	0.00	11	42.31	11	42.31	2	7.69	2
média	0.1379		12.414		11.759		4.2069		2.75862069
	4		360		341		122		80
%	0.441		39.691		37.596		13.451		8.820286659
85%OCP	0.1323		11.907		11.279		4.0353		2.646085998
HRS ENF	0.5292		71.444		112.79		40.353		47.62954796

QP / 24H

POR PLANTÃO

os	Dia do mês	Mínimos		Intermediários		Alta Dependência		Ser Inten
%		n	%	n	%	n	%	n
0.00	2	2	24	3	12	7	28	6
2.94	3	4	13.33	3	10	15	50	5
9.38	4	3	9.38	5	15.63	15	46.88	5
11.76	5	4	12.9	5	16.13	12	38.71	6
12.12	6	5	15.63	5	15.63	12	37.5	6
12.12	7	1	3.7	6	22.22	12	44.44	5
12.50	8	0	0	9	28.13	15	46.88	5
12.90	9	0	0	5	16.13	18	58.06	5
12.12	10	1	3.45	5	17.24	17	58.62	4
10.00	11	0	0	7	22.58	13	41.94	9
9.38	12	0	0	3	10.34	17	58.62	8
9.68	13	1	3.23	3	9.68	17	54.84	7
12.50	14	2	7.69	2	7.69	12	46.15	9
10.34	15	1	3.23	6	19.35	17	54.84	4
9.38	16	1	3.33	4	13.33	15	50	9
6.25	17	2	6.67	2	6.67	16	53.33	8
6.67	18	1	3.85	4	15.38	12	46.15	8
6.45	19	2	6.67	5	16.67	13	43.33	8
6.67	20	1	3.45	7	24.14	14	48.28	6
9.38	21	1	3.57	8	28.57	11	39.29	6
10.34	22	0	0	5	18.52	13	48.15	8
9.38	23	1	6.67	5	33.33	6	40	3
9.38	24	2	7.41	3	11.11	13	48.15	9
9.38	25	1	3.45	6	20.69	12	41.38	10
6.45	26	1	3.45	4	13.79	14	48.28	10
6.06	27	1	3.33	7	23.33	14	46.67	8
6.67	28	0	0	1	8.33	6	50	5
6.45	29	0	0	2	15.38	7	53.85	4
7.69	30	0	0	2	15.38	7	53.85	4
	MÉDIA	1.310345		4.551724		12.82759		6.551724
		38		132		372		190
	%	4.865557		16.90141		47.63124		24.32778
		1.459667		5.070423		14.28937		7.298335
**272.75**		5.838668		30.42254		142.8937		72.98335
73.18								
68.19								
**11.36**								

ni-sivos	Intensivos	
%	n	%
24	3	12
16.67	3	10
15.63	4	12.5
19.35	4	12.9
18.75	4	12.5
18.52	3	11.11
15.63	3	9.38
16.13	3	9.68
13.79	2	6.9
29.03	2	6.45
27.59	1	3.45
22.58	3	9.68
34.62	1	3.85
12.9	3	9.68
30	1	3.33
26.67	2	6.67
30.77	1	3.85
26.67	2	6.67
20.69	1	3.45
21.43	2	7.14
29.63	1	3.7
20	0	0
33.33	0	0
34.48	0	0
34.48	0	0
26.67	0	0
41.67	0	0
30.77	0	0
30.77	0	0

	1.689655172	
	49	
	6.274007682	
	1.882202305	
	33.87964149	**286.0179**
	QP / 24H	76.73861
		71.50448
	POR PLANTÃO	**11.91741**
